# Integrated physical-chemical remediation and human health risk assessment of multi-metal contaminated industrial soils

**DOI:** 10.1007/s11356-026-38177-x

**Published:** 2026-09-02

**Authors:** Lucia Marsich, Alessio Ferluga, Luca Cozzarini, Paolo Bevilacqua

**Affiliations:** 1 University of Trieste Department of Engineering and Architecture: Università degli Studi di Trieste Dipartimento di Ingegneria e Architettura, Trieste, Italy; 2 Research & Innovation Department, MaterialScan S.R.L, Trieste, Italy

**Keywords:** Contaminated soil, Heavy metals, Soil remediation, Mechanical screening, Chemical extraction, Magnetic separation, Health risk assessment

## Abstract

This study presents an integrated remediation strategy and human health risk assessment for multimetal-contaminated industrial soil. The sequential treatment combined mechanical screening, high-intensity magnetic separation (HIMS), and chemical extraction using ethylenediaminetetraacetic acid (EDTA). Initial characterization revealed extreme contamination levels, with cadmium and thallium posing significant noncarcinogenic risks, particularly for children (hazard index = 169.4). Physical separation proved highly effective: Mechanical screening recovered 79% of the soil mass as a cleaner coarse fraction, while HIMS achieved metal removal efficiencies between 73 and 93% for lead, arsenic, and antimony. Subsequent chemical extraction using 45 mM EDTA lowered all hazard indexes below unity for outdoor workers and residential adults. Additionally, the goal for fetal blood lead concentration (10 µg/dL) was reached after treatment with 15 mM EDTA. Despite these achievements, noncarcinogenic risks from cadmium and thallium remained above acceptable thresholds for children, indicating that the remediated soil does not yet meet the stringent requirements for residential land use without further measures. This study demonstrates that combining established physical and chemical techniques within a risk-based framework provides a practical and scientifically robust approach for managing complex multimetal soil contamination. The proposed strategy is viable for industrial or commercial redevelopment, whereas the protection of the most vulnerable populations in residential settings remains a critical challenge.

**Supplementary information:**

The online version contains supplementary material available at https://doi.org/10.1007/s11356-026-38177-x.

## Introduction

Heavy metal contamination of soil represents a serious global concern, particularly in areas affected by industrial activities such as mining, smelting, electroplating, and coal combustion. These processes release persistent toxic elements, including zinc, lead, cadmium, mercury, and arsenic, which accumulate in soils and pose long-term risks to ecosystems and human health (Rikers et al. 1998; Abumaizar and Smith 1999; Dermont et al. 2008; Liao et al. 2016; Wang et al. 2020; Zhang et al. 2022).

Traditional remediation approaches have progressively shifted from containment strategies, such as capping and landfilling, toward multifaceted and integrated approach that increasingly leverages coupled physical–chemical systems to achieve permanent contaminant removal. This strategy balances maximum removal efficiency with the preservation of soil health and biological properties, particularly through the development of synergistic treatment designed to minimize secondary environmental impacts while potentially recovering valuable resources (Griffiths 1995; Dermont et al. 2008; Hashim et al. 2011; Zhang et al. 2022; Yang et al. 2024; Chen and Ding 2025).

Among available remediation options, integrating physical separation and chemical extraction methods offers a promising strategy to enhance the efficiency of heavy metal removal (Dermont et al. 2008; Zhang et al. 2022). The effectiveness of these techniques depends on several factors, including the type and chemical form of the heavy metals, their association within the soil matrix, soil characteristics (i.e., particle size distribution, organic matter content, pH), and the specific washing agents or physical processes employed (Peters 1999; Dermont et al. 2008; Zhang et al. 2022).

*Physical separation* exploits differences in physical characteristics, such as particle size, density, magnetic susceptibility, and surface properties, to concentrate metal-rich fractions into smaller volumes, thereby reducing the amount of soil requiring subsequent treatment. When successfully implemented, physical separation can reduce the volume of contaminated soil requiring further treatment or disposal by up to 90% (Griffiths 1995; Rikers et al. 1998; Peters 1999; Abumaizar and Smith 1999; Mercier et al. 2001; Dermont et al. 2008; Liao et al. 2016; Baragaño et al. 2023). These techniques derive from well-established technologies in the mining and mineral-processing industries, where they are routinely used to extract valuable metal-bearing particles from ores (Wills and Finch 2016). Their advantages include cost-effectiveness, operational simplicity, readily available equipment, and well-documented procedures (Mercier et al. 2001; Dermont et al. 2008).

*Mechanical screening* sorts particles based on their dimensions (Mercier et al. 2001; Dermont et al. 2008). It is particularly effective to separate the fine soil fractions (silt and clay), which typically exhibit higher metal concentrations due to their larger specific surface area that enhances adsorption-type phenomena, from the larger, cleaner sand and gravel fractions. The latter can be reused or returned to the site as backfill, whereas the finer and more contaminated portion is conveyed to additional treatment or disposal (Peters 1999; Dermont et al. 2008; Liao et al. 2016).

*Magnetic separators* can be used to remove magnetic or magnetizable particles containing heavy metals. Low-intensity separators are employed to treat ferromagnetic and highly paramagnetic minerals, whereas weakly paramagnetic minerals can only be effectively recovered using high-intensity magnetic field (Rikers et al. 1998; Mercier et al. 2001; Dermont et al. 2008; Wills and Finch 2016). Numerous heavy metals can be removed from the soil matrix by magnetic separation because they are often associated with magnetic or paramagnetic phases, particularly iron-bearing phases and minerals (Rikers et al. 1998; Mercier et al. 2001; Dermont et al. 2008; Hashim et al. 2011; Begum et al. 2012; Ettler 2016; Tripathy et al. 2017; Zhang et al. 2022, 2025b; Baragaño et al. 2023). Lead and zinc are examples of elements whose minerals are normally diamagnetic. Despite this, they can be removed by high-intensity magnetic separation through an indirect mechanism, because a significant percentage of these elements are associated with ferromagnetic or paramagnetic iron minerals or oxides (Rikers et al. 1998).

Complementary to physical separation, *chemical extraction* involves solubilizing contaminants from soil particles using a washing solution containing extraction agents such as acids, bases, inorganic salts, and chelating agents (Peters 1999; Abumaizar and Smith 1999; Dermont et al. 2008; Liu et al. 2022). Chelating agents form stable, soluble complexes with metal ions, increasing their mobility and removal efficiency.

EDTA (ethylenediaminetetraacetic acid) is among the most effective synthetic chelating agents for soil washing, particularly for cationic metals such as lead, cadmium, copper, and zinc. Its efficiency derives from the formation of highly stable, water-soluble metal–EDTA complexes that remain stable across a broad pH range. Thanks to these strong complexes, EDTA can overcome metal–soil interactions and mobilize contaminants not only from exchangeable, carbonate, and reducible fractions but also, in some cases, from partially oxidizable pools (Dermont et al. 2008; Abumaizar and Smith 1999; Peters 1999; Begum et al. 2012; Oh et al. 2015; Zheng et al. 2022). In contrast to strong mineral acids, such as hydrochloric, sulfuric, and nitric acid, which remove metals through acidolysis and ion exchange and can severely degrade soil structure, EDTA preserves soil physicochemical properties (Cline and Reed 1995; Peters 1999; Oh et al. 2015; Zhang et al. 2022). Mineral acids dissolve mineral solids and organic matter and leave the soil at very low pH, necessitating extensive post-treatment neutralization (Peters 1999; Abumaizar and Smith 1999; Ko et al. 2006; Lin et al. 2012; Liu et al. 2022; Zhang et al. 2022; Zheng et al. 2022). EDTA, on the other hand, preserves soil physicochemical properties and maintains a moderate final pH (Peters 1999; Oh et al. 2015; Zhang et al. 2022). Although biodegradable chelators, such as EDDS ([S,S]-ethylenediaminedisuccinic acid) and GLDA (N,N-bis(carboxymethyl)-l-glutamic acid), are increasingly explored as environmentally friendlier alternatives, their metal-removal performance is generally lower. EDTA typically achieves higher metal removal due to its stronger complexation ability and wider operational pH range. This superior extraction efficiency has also been confirmed under realistic field conditions. A further advantage is its recyclability: EDTA can be recovered from washing effluents through electrodeposition or precipitation and reused multiple times, making it suitable for industrial-scale applications (Dermont et al. 2008; Hashim et al. 2011; Zhang et al. 2022; Zheng et al. 2022). Finally, EDTA is significantly more cost-effective than biodegradable variants such as EDDS, which are still too expensive for widespread field remediation (Chen et al. 2022; Zhang et al. 2022).

The integration of physical separation and EDTA-assisted chemical extraction offers a cost-effective approach to heavy metal remediation, which can also be scaled up and implemented in pilot-scale or full-scale industrial treatment plants (Peters 1999; Dermont et al. 2008; Lin et al. 2012; Liao et al. 2016).

However, most remediation studies focus primarily on contaminant removal efficiency, without comprehensive risk-based assessment across multiple exposure scenarios. (Abumaizar and Smith 1999; Mercier et al. 2001; Ko et al. 2006; Begum et al. 2012; Liao et al. 2016; Baragaño et al. 2023; Ganesh et al. 2026). To address this limitation, incorporating a human health risk assessment provides a quantitative basis for evaluating treatment effectiveness across different exposure scenarios and is increasingly recognized as an essential component of environmental pollution research (Oh et al. 2015; Liu et al. 2022; Chen and Ding 2025; Zhang et al. 2025a, c).

Human health risk assessment is a critical tool for estimating the potential adverse health effects in humans exposed to heavy metal contaminants present in soil. It helps to quantify the risk and can be used to set goals and priorities for environmental protection (Li et al. 2019; Abouian Jahromi et al. 2020; Soltanian et al. 2022; Ramires et al. 2024, 2023; Siddig et al. 2025). This assessment is particularly vital in areas influenced by anthropogenic activities, such as industrial processes, mining, agriculture, traffic, and waste disposal, where soil metal concentrations can be elevated. The human health exposure risk assessment methodologies are based on the principles developed by the United States Environmental Protection Agency (USEPA) (USEPA 1989, 1991, 2002, 2003, 2011).

**Fig. 1:**
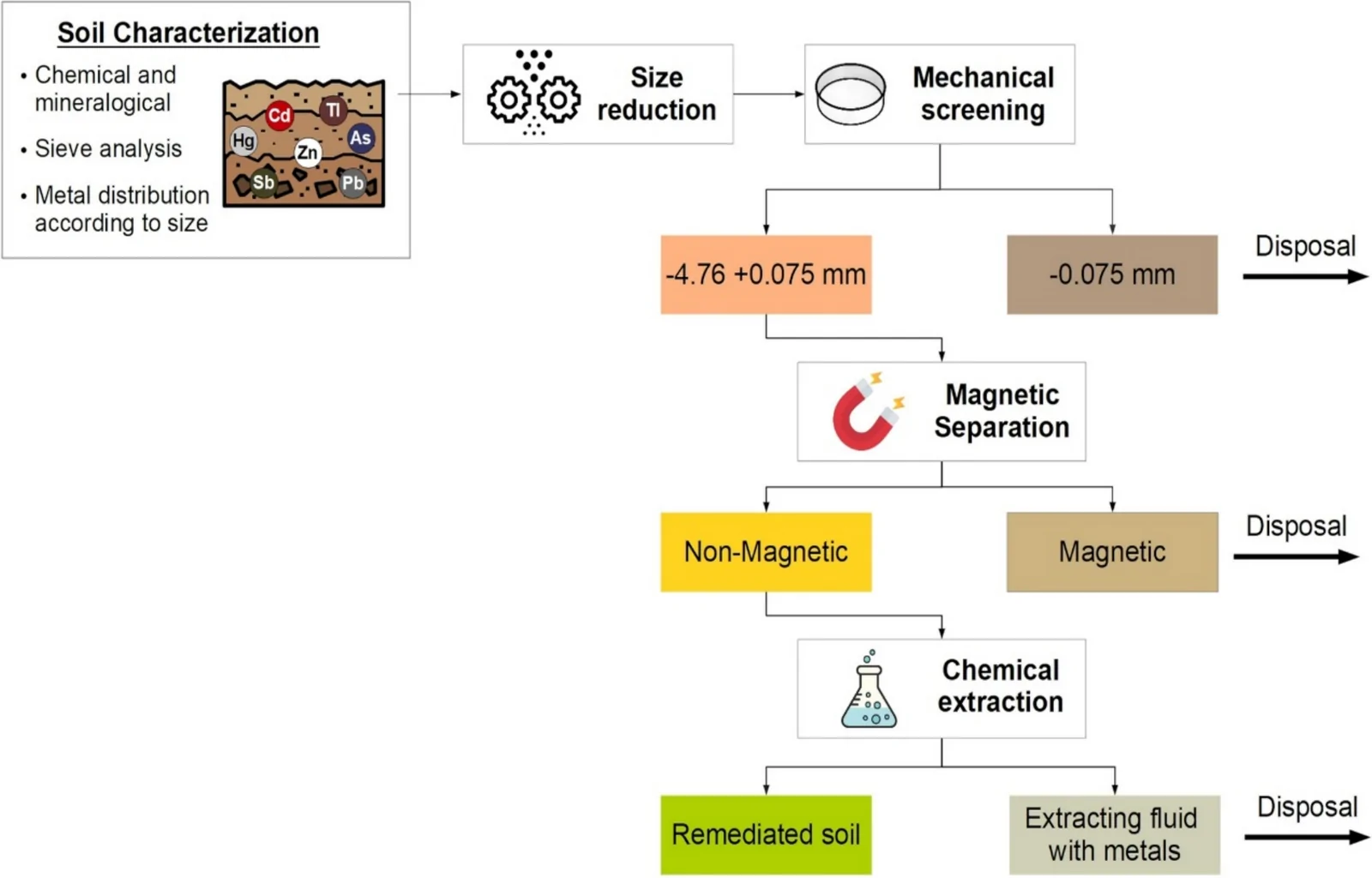
Remediation process scheme adopted in this study. The diagram summarizes the sequential steps used to (i) characterize the multimetal contaminated soil, (ii) apply physical separation (mechanical screening and high-intensity magnetic separation), and (iii) perform EDTA-assisted chemical extraction at increasing concentrations (15–75 mM)

Therefore, this study presents an integrated physical and chemical remediation strategy applied to industrial soil contaminated with multiple heavy metals, including zinc, lead, cadmium, mercury, iron, copper, antimony, arsenic, and selenium. The treatment sequence combines mechanical screening to isolate the most contaminated fine fractions, high-intensity magnetic separation to further remove metal-rich particles, and EDTA-assisted chemical extraction to mobilize residual contaminants (Fig. 1). Complementing the technical evaluation, a comprehensive human health risk assessment based on USEPA methodologies (USEPA 1989, 2003) was conducted to quantify how each remediation step influences both carcinogenic and noncarcinogenic risks across outdoor worker, adult, and child exposure scenarios. This integrated approach allows not only the assessment of contaminant removal performance but also the identification of exposure conditions for which additional management actions may be required. Overall, the study provides a risk-based framework for evaluating multistep remediation strategies in complex contamination settings, contributing to the development of more efficient, economically viable, and adaptive remediation practices for industrial sites.

## Materials and methods

### Characterization of the soil

A 200-kg sample of multiheavy metal–contaminated soil was collected from a smelting industrial site in Italy. After coning and quartering in accordance with ASTM C702, the soil sample was divided into two representative samples. One was used for soil characterization, while a 20-kg portion underwent a multistage remediation process.

The amounts of antimony (Sb), arsenic (As), cadmium (Cd), cobalt (Co), chromium (Cr), iron (Fe), mercury (Hg), nickel (Ni), lead (Pb), copper (Cu), selenium (Se), tin (Sn), thallium (Tl), titanium (Ti), vanadium (V), and zinc (Zn) were determined in a 1-kg representative sample of the original soil after coning and quartering. Soil digestion and elemental determination were performed in accordance with UNI EN 13657, UNI EN 16170, and ISO 22036. A microwave reaction system (Ultrawave 3, Milestone, Sorisole, BG) was used for the digestion treatment of the soil samples. The digestion was performed at 40 bar and 165 °C for 30 min. The digested sample was filtered on a 0.45-µm filter, and the metal concentrations were measured using an inductively coupled plasma optical emission spectrometer (ICP-OES 5900, Agilent Technologies, Santa Clara, CA). In compliance with ISO 22036, blank test solutions prepared during the extraction process were systematically used, and the instrument calibration was verified using the multielement calibration standards ICUS-5619 and ICUS-5620 (Agilent Technologies, Santa Clara, CA).

Particle density of soil samples was determined using pycnometer method by fluid displacement in accordance with UNI EN ISO 17892-3. Particles with a size larger than 4 mm were crushed to meet the requirements of the standard.

XRD analysis was performed to obtain mineralogical information on the contaminated soil. Specimens were pulverized using a manual vibratory disc mill (Herzog, Osnabrück, Germany) with an iron jar to pass through a sieve with 0.150-mm aperture. The XRD pattern was collected using a Bruker D5005 diffractometer operating at 40 kV and 30 mA, with Cu Kα radiation (*E* = 8.04 keV; *λ* = 1.5406 Å), over a 2θ range of 5–70° with step size 0.05° and a count time of 10 s per step. Profex software version 5.4.1 (Doebelin and Kleeberg 2015) and the Crystallography Open Database (COD) were used to analyze the XRD pattern.

The particle size analysis was performed by means of sieve analysis on a representative soil sample, obtained by coning and quartering. A Weston screen machine with a 600-mm sieve diameter was used to divide the soil into nine size fractions. The soil was dry-sieved through sieves with apertures of 36 mm, 19 mm, 4.76 mm, and 0.841 mm. Subsequently, the undersize fraction from the dry-sieving was wet-sieved through sieves with apertures of 0.255 mm, 0.150 mm, 0.075 mm, and 0.010 mm.

After the sieve analysis, the distribution of heavy metal concentrations according to particle size was evaluated.

Physicochemical and mineralogical characteristics of the soil were also determined after each physical separation step and after chemical extraction.

Optical microscopy images were collected by a DinoLite AM4515ZT microscope.

### Physical separation methods

A representative 20-kg nonsieved soil sample was obtained by coning and quartering and then reduced to a granulate by jaw crushers and cylindrical mills (SAIMA—Speciali Apparecchiature Industriali Meccaniche e Affini, Milano, Italy), producing a fraction with particle sizes below 4.76 mm.

*Mechanical screening* was performed on this material: The soil was wet-sieved through sieves with apertures of 1.4 mm, 0.425 mm, and 0.075 mm. The finest fraction (< 0.075 mm), which previous analyses had identified as the most contaminated, was separated and designated for disposal.

*The magnetic separation* was applied on the −4.76 + 0.075 mm particle size fraction using a laboratory-scale high-intensity rare-earth magnetic roll separator (Gauss magneti S.r.l., Brescia, Italy) equipped with 100-mm neodymium-boron-iron roll operating at magnetic induction of 2 T. A 14-kg sample of soil within this size range was divided into seven subsamples for processing. Prior to magnetic treatment, each subsample was dry-sieved through sieve with 1.0-mm apertures. Undersize and oversize fractions were magnetically separated using belt speeds of 0.5 m/s and 0.38 m/s, respectively, to ensure optimal separation efficiency.

### Chemical extraction

The nonmagnetic fraction was then subjected to chemical extraction. During each chemical extraction, 40 g of material was processed in 400 mL of extracting solution for 10 min. Mixing was ensured by a Jar Test apparatus at a constant speed of 160 rpm. The treated soil was separated from the extracting solution by filtration through a 38-µm sieve and subsequently rinsed with water and filtered again to remove residual EDTA and chelated metal complexes from the soil matrix.

EDTA (ethylenediaminetetraacetic acid) aqueous solutions with concentrations between 15 and 75 mM were tested.

A control test was conducted by processing the nonmagnetic fraction under identical conditions (40 g in 400 mL, 10 min, 160 rpm) without EDTA, in order to quantify metal release due solely to mixing and contact with water.

All tests were carried out in duplicate.

### Human health risk assessment

The health risks for humans exposed to contaminated soil have been assessed after each remediation treatment using the method developed by the United States Environmental Protection Agency (USEPA) (USEPA).

Human exposure was estimated by considering three pathways: ingestion, dermal contact, and inhalation. In this study, both nonresidential and residential exposure scenarios were considered. The average daily intake, expressed in mg kg^−1^ day^−1^, of heavy metals through ingestion (${D}_{\mathrm{i}\mathrm{n}\mathrm{g}}$), dermal contact (${D}_{\mathrm{d}\mathrm{e}\mathrm{r}\mathrm{m}}$), and inhalation (${D}_{\mathrm{i}\mathrm{n}\mathrm{h}}$) was calculated using Eqs. (1), (2), and (3) (USEPA 1989).

1 $$\mathrm{D}_{\mathrm{i}\mathrm{n}\mathrm{g}}=\frac{{\mathrm{C}}_{\mathrm{M}}\times \mathrm{I}\mathrm{R} \times \mathrm{E}\mathrm{F}\times \mathrm{E}\mathrm{D}\times \mathrm{C}\mathrm{F}}{\mathrm{B}\mathrm{W}\times \mathrm{A}\mathrm{T}}$$

2 $$\mathrm{D}_{\mathrm{d}\mathrm{e}\mathrm{r}\mathrm{m}}=\frac{{\mathrm{C}}_{\mathrm{M}}\times \mathrm{S}\mathrm{A} \times \mathrm{A}\mathrm{F}\times \mathrm{A}\mathrm{B}\mathrm{S} \times \mathrm{E}\mathrm{F}\times \mathrm{E}\mathrm{D}\times \mathrm{C}\mathrm{F}}{\mathrm{B}\mathrm{W}\times \mathrm{A}\mathrm{T}}$$

3 $$\mathrm{D}_{\mathrm{i}\mathrm{n}\mathrm{h}}=\frac{{\mathrm{C}}_{\mathrm{M}}\times \mathrm{I}\mathrm{n}\mathrm{h}\mathrm{R} \times \mathrm{E}\mathrm{F}\times \mathrm{E}\mathrm{D}}{\mathrm{P}\mathrm{E}\mathrm{F}\times \mathrm{B}\mathrm{W}\times \mathrm{A}\mathrm{T}}$$

Parameter definitions and values related to the abovementioned scenarios are reported in Table 1.

**Table 1:** Parameter definitions and values used for the calculation of the intake of heavy metals through ingestion, dermal contact, and inhalation for residential (adult and child) and nonresidential scenarios (outdoor worker) (USEPA 2002, 2011)

Parameter	Definition		Value			Unit
			Not residential	Residential		
			Outdoor worker	Adult	Child	
$C_M$	Heavy metal concentration		-			mg kg^−1^
IR	Ingestion rate		100	100	200	mg day^−1^
InhR	Inhalation rate		20			mg day^−1^
EF	Exposure frequency		225	365	365	Days year^−1^
ED	Exposure duration		25	30	6	Year
CF	Conversion factor		10^–6^			kg mg^−1^
BW	Body weight		70	70	15	kg
AT	Averaging time	Noncarcinogenic risk	9125	10,950	2190	Days
		Carcinogenic risk	25,500			Days
SA	The exposed skin surface area		3300	5700	2800	cm^2^
AF	Adherence factor		0.2	0.07	0.2	mg cm^−3^
ABS	Dermal absorption factor		Chemical-specific value			-
PEF	Particle emission factor		1.36 10^9^			m^3^ kg^−1^

The nonresidential scenario focuses on outdoor workers, exposed to soil during activities such as digging and landscaping. The residential scenario assumes long-term exposure and higher ingestion rates and soil contact activities for children.

For all heavy metals, the dermal absorption factor (ABS) is 0.01, except for cadmium (0.001) and arsenic (0.03).

To estimate noncarcinogenic risk, the hazard quotient (HQ) was calculated by dividing the average daily intake through ingestion, dermal contact, and inhalation by the respective reference dose (RfD_i_) (Eqs. (4), (5), and (6)).

4 $${\mathrm{H}\mathrm{Q}}_{\mathrm{i}\mathrm{n}\mathrm{g}}=\frac{{\mathrm{D}}_{\mathrm{i}\mathrm{n}\mathrm{g}}}{{\mathrm{R}\mathrm{f}\mathrm{D}}_{0}}$$

5 $${\mathrm{H}\mathrm{Q}}_{\mathrm{d}\mathrm{e}\mathrm{r}\mathrm{m}}=\frac{{\mathrm{D}}_{\mathrm{d}\mathrm{e}\mathrm{r}\mathrm{m}}}{{\mathrm{R}\mathrm{f}\mathrm{D}}_{\mathrm{A}\mathrm{B}\mathrm{S}}}=\frac{{\mathrm{D}}_{\mathrm{d}\mathrm{e}\mathrm{r}\mathrm{m}}}{{\mathrm{R}\mathrm{f}\mathrm{D}}_{0}\times {\mathrm{A}\mathrm{B}\mathrm{S}}_{\mathrm{G}\mathrm{I}}}$$

6 $${\mathrm{H}\mathrm{Q}}_{\mathrm{i}\mathrm{n}\mathrm{h}}=\frac{{\mathrm{D}}_{\mathrm{i}\mathrm{n}\mathrm{h}}}{{\mathrm{R}\mathrm{f}\mathrm{D}}_{\mathrm{i}\mathrm{h}\mathrm{n}}}=\frac{{\mathrm{D}}_{\mathrm{i}\mathrm{n}\mathrm{h}}\times \mathrm{B}\mathrm{W}\times \mathrm{A}\mathrm{T}}{{\mathrm{R}\mathrm{f}\mathrm{D}}_{\mathrm{C}\mathrm{i}}\times \mathrm{I}\mathrm{n}\mathrm{h}\mathrm{R}\times \mathrm{E}\mathrm{F}\times \mathrm{E}\mathrm{D}}$$

The chronic oral reference dose (RfD_0_), the chronic inhalation reference concentration (RfD_ci_), and the fraction of contaminant absorbed in the gastrointestinal tract (ABS_GI_) are chemical-specific and are reported in regional screening level (RSL) tables (USEPA 2015).

When assessing multiple exposure routes and/or multiple substances, the hazard index (HI) is utilized. In this study, the combined noncarcinogenic risk of a single metal through the three exposure pathways (ingestion, dermal contact, and inhalation) was estimated by summing the HQs for each pathway (Eq. (7)).

7 $${\mathrm{H}\mathrm{I}}_{\mathrm{M}}=\sum {\mathrm{H}\mathrm{Q}}_{\mathrm{M}}= {\mathrm{H}\mathrm{Q}}_{\mathrm{M},\mathrm{i}\mathrm{n}\mathrm{g}}+ {\mathrm{H}\mathrm{Q}}_{\mathrm{M},\mathrm{d}\mathrm{e}\mathrm{r}\mathrm{m}}+{\mathrm{H}\mathrm{Q}}_{\mathrm{M},\mathrm{i}\mathrm{n}\mathrm{h}}$$

An HQ < 1 and HI < 1 indicate no significant risk of adverse noncarcinogenic health effects. Conversely, HQ and HI greater than 1 indicate an unacceptable risk of adverse noncarcinogenic health effects.

The carcinogenic health risk (CR) represents the incremental lifetime probability of an individual developing cancer as a result of exposure to a carcinogenic chemical (USEPA 1989). It was assessed for arsenic, cadmium, and nickel, as these elements are classified as carcinogenic by the Integrated Risk Information System (IRIS) (USEPA 2013): Arsenic is categorized as group A (known human carcinogen), while cadmium and nickel are classified as group B1 (probable human carcinogens). Arsenic is considered carcinogenic via ingestion, dermal exposure, and inhalation, while cadmium and nickel are considered carcinogenic only via inhalation. The CR was calculated by multiplying the average daily intake (*D*_i_) by the corresponding slope factor (SF) as shown in Eqs. (8) and (9).

8 $${\mathrm{C}\mathrm{R}}_{\mathrm{i}}=\mathrm{D}_{\mathrm{i}}\times {\mathrm{S}\mathrm{F}}_{\mathrm{i}}$$

9 $$\mathrm{C}\mathrm{R}=\sum {\mathrm{C}\mathrm{R}}_{\mathrm{i}}$$

The cancer slope factor (SF_i_) values are reported in Table 2.

**Table 2:** Cancer slope factor (SF_i_) values of arsenic, cadmium, and nickel

Parameter	Definition	Value			Unit
		Arsenic	Cadmium	Nickel	
SF_ing_	Cancer slope factor—ingestion	1.5	n.a	n.a	mg^−1^ kg day
SF_d_	Cancer slope factor—dermal contact	1.5	n.a	n.a	mg^−1^ kg day
SF_inh_	Cancer slope factor—inhalation	15.05	1.8	0.24	mg^−1^ kg day

A cancer risk value lower than 1 × 10^–6^ is considered negligible, whereas a CR above 1 × 10^−4^ is deemed unacceptable. A CR within a range from 1 × 10^−6^ to 1 × 10^−4^ indicates an acceptable or tolerable risk (USEPA 1991; Fowle and Dearfield 2000).

The USEPA proposes an alternative approach to assessing risks associated with adult exposure to lead in soil, because a reference dose (RfD) value for lead is currently unavailable. Blood lead concentration can be correlated with both exposure levels and adverse health effects. This methodology specifically focuses on estimating fetal blood lead concentration in women exposed to lead-contaminated soils. Furthermore, this approach offers tools for evaluating the risks of elevated blood lead concentrations among exposed adults (USEPA 2003).

The blood lead concentrations for exposed adults (PbB_adult,central_) can be estimated as the sum of the blood lead concentration in the absence of site exposure (PbB_adult,0_) and an expected site-related increase (Eq. (10)).

10 $${\mathrm{P}\mathrm{b}\mathrm{B}}_{\mathrm{a}\mathrm{d}\mathrm{u}\mathrm{l}\mathrm{t},\mathrm{c}\mathrm{e}\mathrm{n}\mathrm{t}\mathrm{r}\mathrm{a}\mathrm{l}}={\mathrm{P}\mathrm{b}\mathrm{B}}_{\mathrm{a}\mathrm{d}\mathrm{u}\mathrm{l}\mathrm{t},0}+\frac{\mathrm{P}\mathrm{b}\mathrm{S}\times \mathrm{B}\mathrm{K}\mathrm{S}\mathrm{F}\times {\mathrm{I}\mathrm{R}}_{\mathrm{s}}\times {\mathrm{A}\mathrm{F}}_{\mathrm{s}}\times {\mathrm{E}\mathrm{F}}_{\mathrm{s}}}{\mathrm{A}\mathrm{T}}$$

The expected fetal blood lead concentrations are proportional to the adult blood lead concentrations (Eq. (11)).

11 $${\mathrm{P}\mathrm{b}\mathrm{B}}_{\mathrm{f}\mathrm{e}\mathrm{t}\mathrm{a}\mathrm{l},0.95}={\mathrm{P}\mathrm{b}\mathrm{B}}_{\mathrm{a}\mathrm{d}\mathrm{u}\mathrm{l}\mathrm{t},\mathrm{c}\mathrm{e}\mathrm{n}\mathrm{t}\mathrm{r}\mathrm{a}\mathrm{l}}\times {\mathrm{G}\mathrm{S}\mathrm{D}}_{\mathrm{i},\mathrm{a}\mathrm{d}\mathrm{u}\mathrm{l}\mathrm{t}}^{1.645}\times {R}_{\mathrm{f}\mathrm{e}\mathrm{t}\mathrm{a}\mathrm{l}/\mathrm{m}\mathrm{a}\mathrm{t}\mathrm{e}\mathrm{r}\mathrm{n}\mathrm{a}\mathrm{l}}<{\mathrm{P}\mathrm{b}\mathrm{B}}_{\mathrm{f}\mathrm{e}\mathrm{t}\mathrm{a}\mathrm{l},0.95,\mathrm{g}\mathrm{o}\mathrm{a}\mathrm{l}}$$

The USEPA (USEPA 2003) considered 10 μg/dL a fetal blood lead level of concern, because fetuses and neonates are a highly sensitive population with respect to the adverse effects of lead on development.

Parameter definitions and values for blood lead concentrations calculation are reported in Table 3.

**Table 3:** Parameter definitions and values used for lead risk assessment

Parameter	Definition	Value	Unit
PbB_adult,0_	Typical blood lead concentration in adults	1.7	μg dL^−1^
PbS	Soil lead concentration	-	mg kg^−1^
BKSF	Biokinetic slope factor	0.4	μg dL^−1^
IR_s_	Intake rate of soil	0.05	g day^−1^
AF_s_	Absolute gastrointestinal absorption fraction	0.12	-
EF_s_	Exposure frequency	219	Day year^−1^
AT	Averaging time	365	Day
GDS_i,adult_	Estimated value of the individual geometric standard deviation	1.8	-
R_fetal/maternal_	Constant of proportionality between fetal blood lead concentration at birth and maternal blood lead concentration	0.9	-
PbB_fetal,0.95 goal_	Goal for the 95th percentile blood lead concentration among fetuses born to women having exposures to the specified site soil concentration	10	μg dL^−1^

## Results

### Soil characterization and metal distribution according to particle size

The original soil, collected from a smelting industrial site in Italy, exhibited extensive multimetal contamination (Table 4).

**Table 4:** Contaminant concentrations in the original soil sample collected from a smelting industrial site

Element	Concentration in the original soil (mg/kg)
Antimony (Sb)	63.9 ± 7.5
Arsenic (As)	159 ± 18
Cadmium (Cd)	1140 ± 105
Chromium (Cr)	84.9 ± 9.9
Cobalt (Co)	11.6 ± 0.8
Copper (Cu)	759 ± 56
Iron (Fe)	25,695 ± 2107
Lead (Pb)	13,800 ± 1587
Mercury (Hg)	54.6 ± 8.6
Nickel (Ni)	24.3 ± 1.6
Selenium (Se)	64.1 ± 4.4
Thallium (Tl)	10.9 ± 0.8
Tin (Sn)	51.6 ± 6.0
Titanium (Ti)	374 ± 65.5
Vanadium (V)	20.5 ± 2.1
Zinc (Zn)	29,500 ± 2036

Iron, lead, and zinc were present at extremely elevated levels (25,695 mg/kg, 13,800 mg/kg, and 29,500 mg/kg respectively). Cadmium (1140 mg/kg), mercury (54.6 mg/kg), copper (759 mg/kg), antimony (63.9 mg/kg), arsenic (159 mg/kg), and selenium (64.1 mg/kg) were also detected at high concentrations. Each of these elements exceeded the Italian soil contamination threshold levels for industrial sites (D.lgs. n. 152/2006): Specifically, cadmium, mercury, lead, zinc, arsenic, and selenium were 76, 11, 14, 20, 3, and 4 times above their respective regulatory limits.

**Fig. 2:**
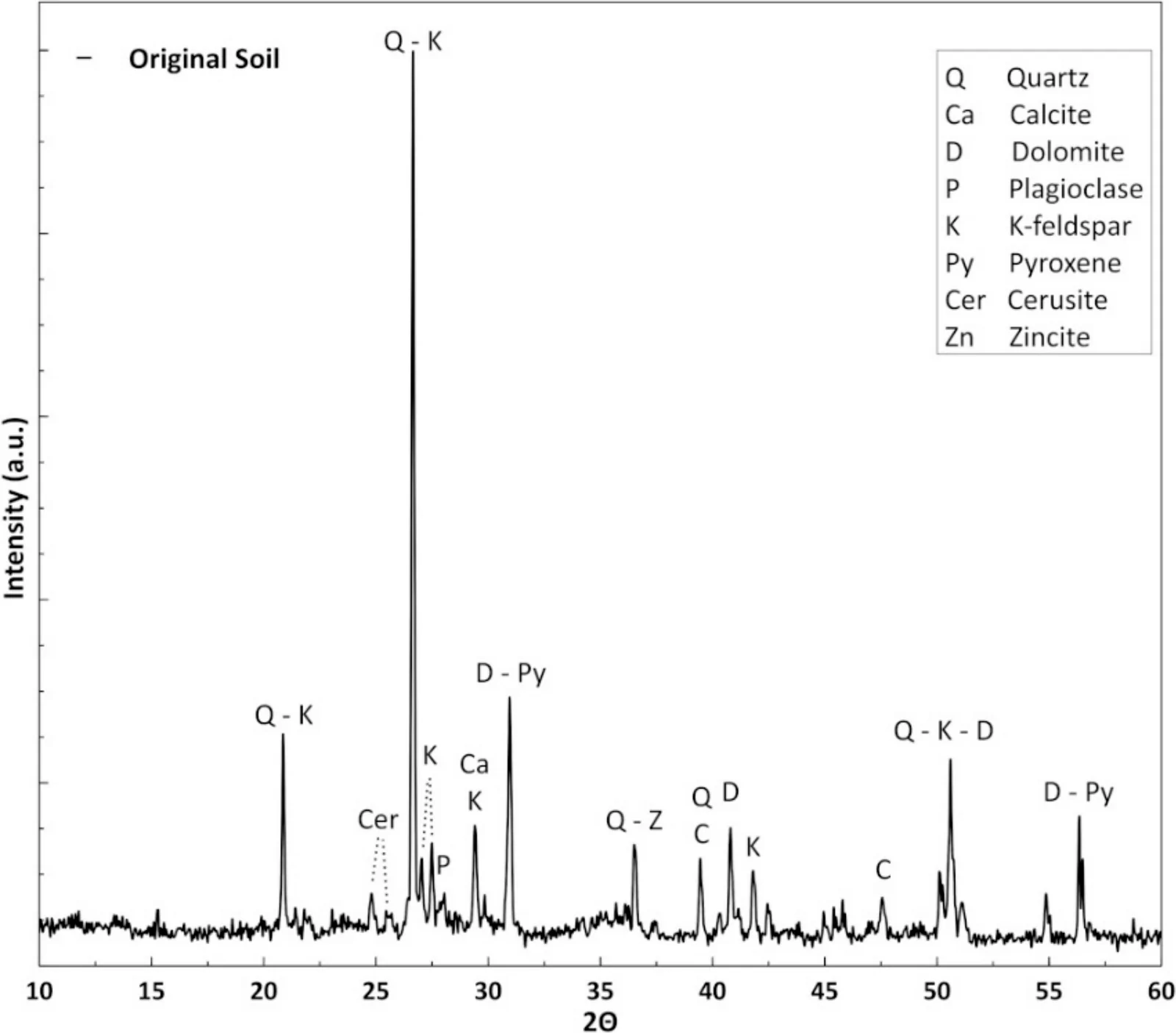
X-ray diffractogram of the original soil

The XRD pattern (Fig. 2) revealed that the original soil, having a bulk density of 2.68 g/cm^3^, exhibited a heterogeneous and mineralogically complex matrix. Quartz (diffraction angle 26.7°—SiO_2_) was identified as the primary constituent, accompanied by calcite (CaCO_3_), dolomite (CaMg(CO_3_)_2_), plagioclase (tectosilicate with Ca, Al, Na), K-feldspar (potassium-aluminum silicate), and pyroxenes, specifically enstatite (Mg_2_Si_2_O_6_) and ferrosilite (Fe_2_Si_2_O_6_).

**Fig. 3:**
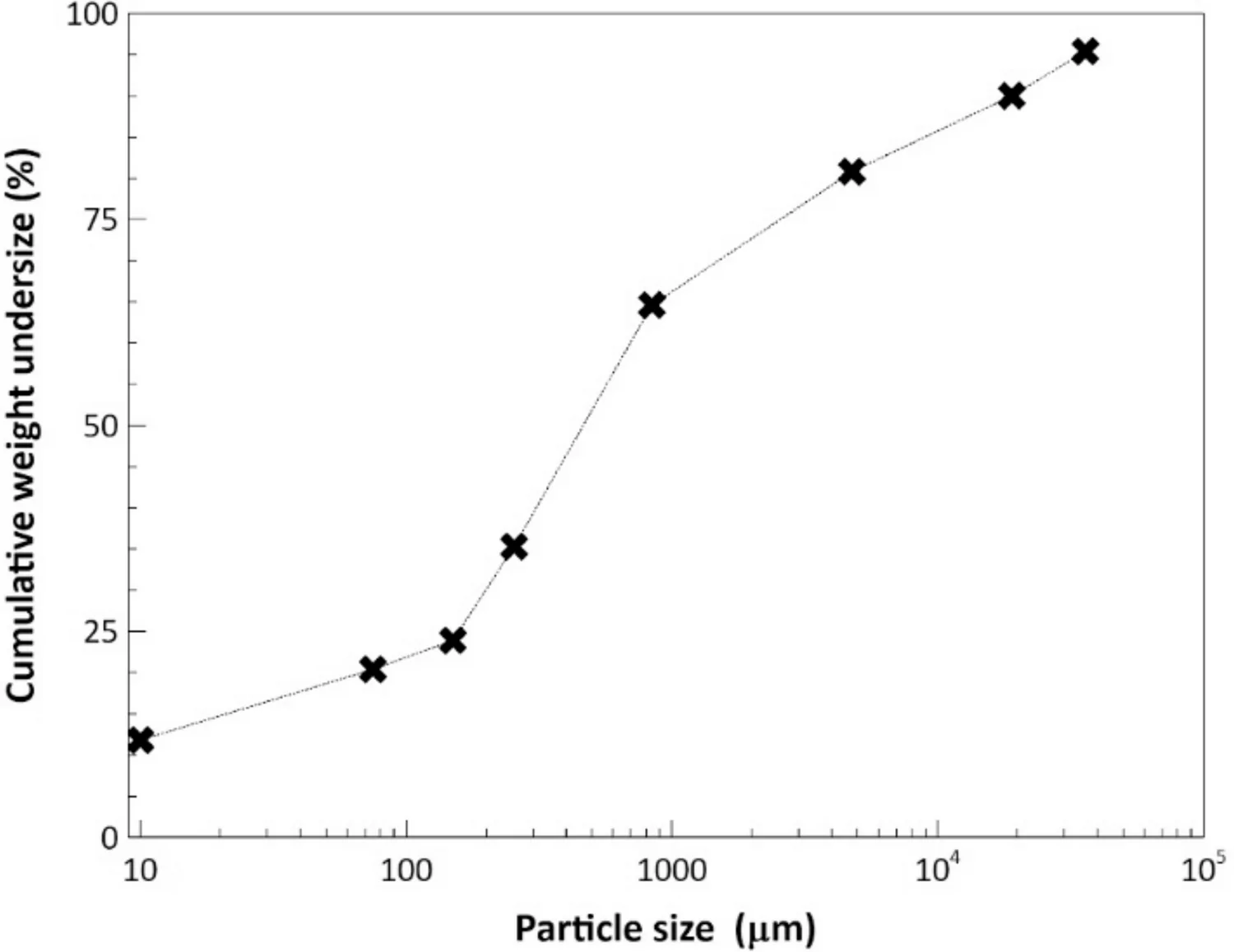
Sieve analysis: particle size distribution

The particle size analysis was performed by means of sieve analysis as a pretreatment step to obtain information about the size and size distribution of particles in the soil. The material was separated into nine fractions of progressively decreasing size: + 36 mm, −36 + 19 mm, −19 + 4.76 mm, −4.76 + 0.841 mm, −0.841 + 0.255 mm, −0.255 + 0.150 mm, −0.150 + 0.075 mm, −0.075 + 0.010 mm, and −0.010 mm, which corresponded to 4.6%, 5.4%, 9.2%, 16.1%, 29.4%, 11.4%, 3.5%, 8.6%, and 11.8% of the original soil sample (Fig. 3). The particle size distribution indicated that the soil predominantly consisted of coarse sand containing a noticeable proportion of finer particles, consistent with particle size ranges typically associated with coarse sand containing fines (ASTM D2487).

**Fig. 4:**
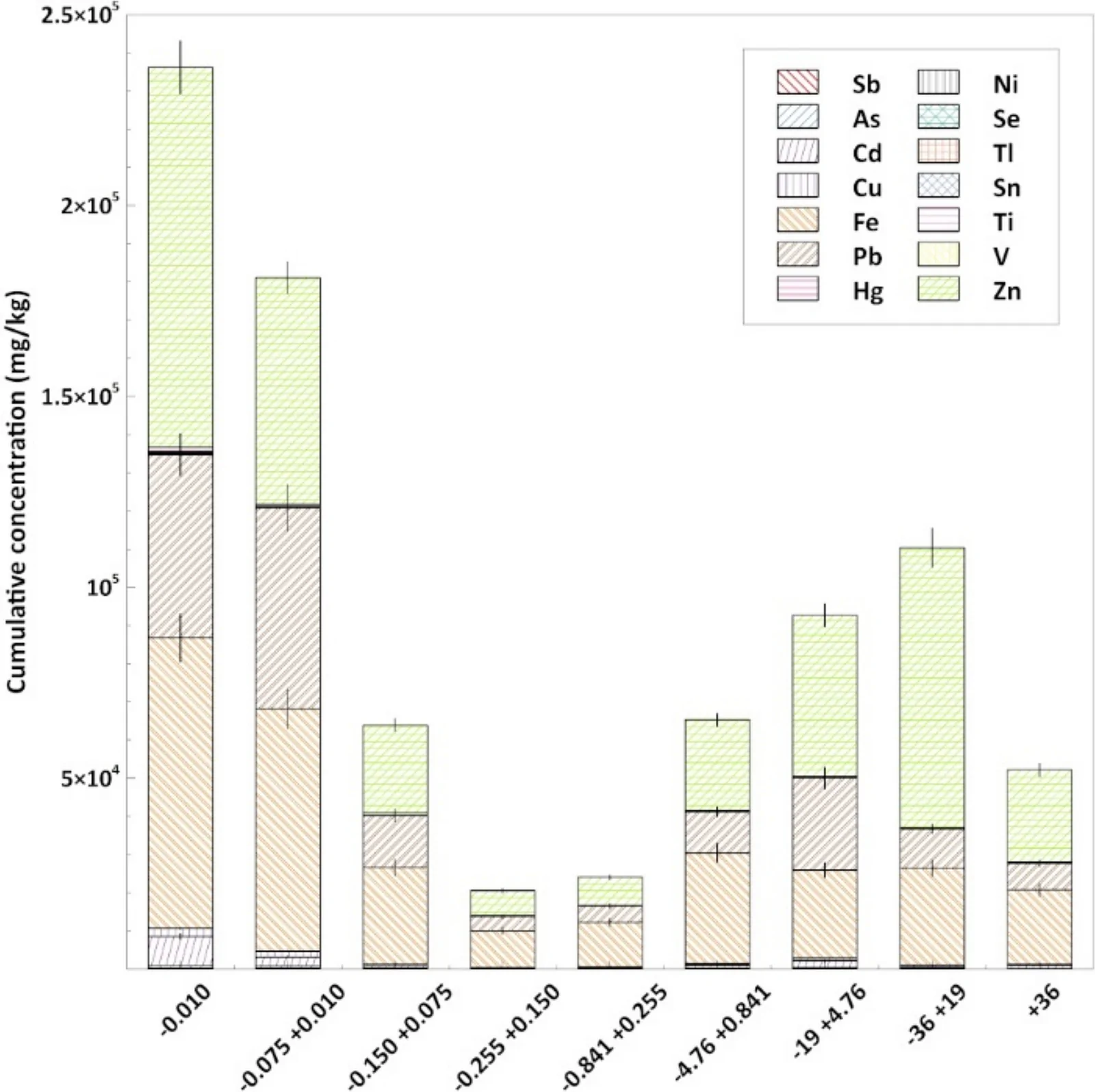
Cumulative representation of the heavy metal concentrations according to particle size

The distribution of heavy metals across the different particle size fractions, illustrated in Fig. 4, showed that all fractions contained extensive contaminant loads, with total metal concentrations ranging from 20 to 240 g per kg.

The finest fractions (−0.010 mm and + 0.010–0.075 mm) exhibited the highest contamination levels, reaching 236 g/kg and 181 g/kg, respectively. In particles smaller than 10 µm, metal concentrations were 3 to 7 times greater than in the original soil, whereas particles in the 10–75 µm range exhibited concentrations 2 to 4 times higher.

In contrast, fractions larger than 0.075 mm generally displayed metal concentrations similar to or lower than those of the original soil. An exception occurred in the + 4.76–19 mm fraction, where antimony and tin were notably enriched. Additional enrichment patterns were observed in coarser fractions: Tin and zinc accumulated in the + 19–36 mm fraction, while thallium concentrations were highest in particles exceeding 36 mm.

### Performance of physical separation process

#### Mechanical screening

Mechanical screening was performed on a representative sample obtained by reducing the nonsieved soil to a granulate with particle sizes below 4.76 mm. The material was divided into two size fractions: −0.075 mm and −4.76 + 0.075 mm.

The fraction with particles smaller than 0.075 mm represented 21% of the sample mass. In this fraction, all contaminants were 2 to 7 times more concentrated than in the fraction between 0.075 and 4.76 mm (Table 5).

**Table 5:** Metal concentrations in the fraction −4.76 + 0.075 mm and −0.075 mm

	Fraction −4.76 + 0.075 mm	Fraction −0.075 mm
Mass %	79.2%	20.8%
Specific gravity (g/cm^3^)	2.67 ± 0.01	2.68 ± 0.05
Antimony (mg/kg)	46.9 ± 5.5	161 ± 19
Arsenic (mg/kg)	83.6 ± 9.2	562 ± 62
Cadmium (mg/kg)	584 ± 54	3560 ± 328
Chromium (mg/kg)	45 ± 5	103 ± 12
Cobalt (mg/kg)	9.1 ± 0.6	21.0 ± 1.4
Copper (mg/kg)	358 ± 27	2057 ± 152
Iron (mg/kg)	19,778 ± 1622	56,300 ± 4617
Lead (mg/kg)	8175 ± 940	42,200 ± 4853
Mercury (mg/kg)	35.7 ± 5.6	175 ± 28
Nickel (mg/kg)	21.0 ± 1.4	50 ± 3
Selenium (mg/kg)	55.1 ± 3.8	118 ± 8
Thallium (mg/kg)	8.4 ± 0.6	35.7 ± 2.7
Tin (mg/kg)	24.5 ± 2.9	96 ± 11
Titanium (mg/kg)	278 ± 49	738 ± 129
Vanadium (mg/kg)	17.1 ± 1.7	51.5 ± 5.3
Zinc (mg/kg)	17,497 ± 1207	73,624 ± 5080

The fraction between 0.075 and 4.76 mm (79% of the sample mass) was less contaminated than the original soil. The concentrations of antimony, lead, mercury, and zinc decreased by approximately 1.5 times, and the concentrations of arsenic, cadmium, copper, chromium, and tin were two times lower than those in the original soil.

All elements, with the exception of copper and thallium, exceeded the Italian soil contamination threshold levels for industrial sites (D.lgs. n. 152/2006). Specifically, cadmium, lead, mercury, and zinc concentrations were still 39, 8, 7, and 12 times higher than their respective regulatory limits.

**Fig. 5:**
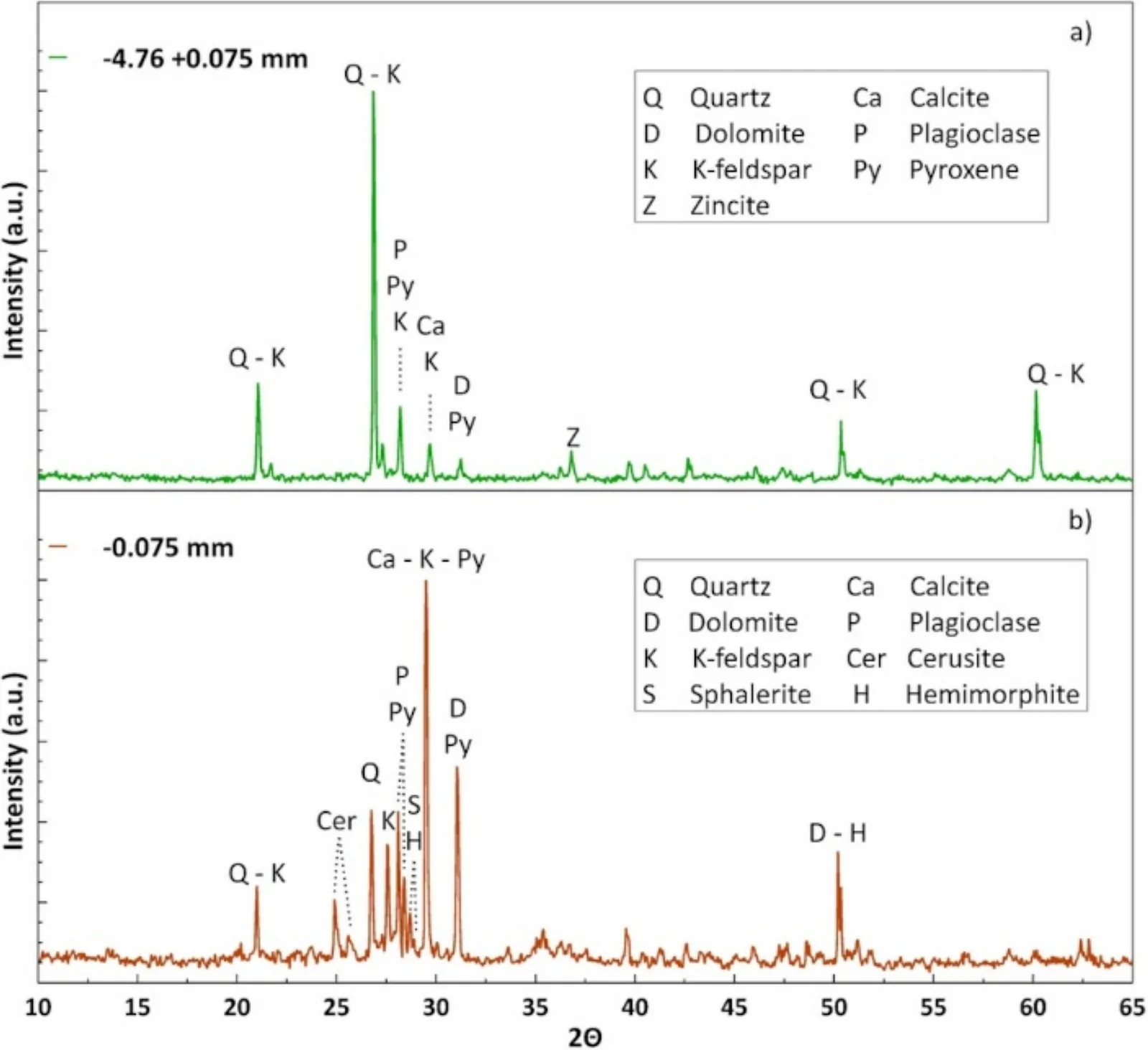
XRD pattern of the fractions obtained after physical separation: **a** −4.76 + 0.075 mm and **b** −0.075 mm

The X-ray diffraction (XRD) pattern of the −0.075 mm fraction (Fig. 5) revealed several distinct diffraction peaks. The most intense peaks, observed at 2θ values of 29.5° and 31°, were consistent with the presence of calcite and dolomite, with the presence of K-feldspar, pyroxenes (specifically ferrosilite). Further mineralogical analysis of this fraction also identified quartz (SiO_2_), plagioclase, and cerussite (PbCO_3_), along with minor amounts of hemimorphite (zinc silicate) and sphalerite (ZnS). A bulk density of 2.68 g/cm^3^ for this fraction is consistent with the presence of calcite and dolomite as their main components.

The mineralogical analysis of −4.76 + 0.075 mm fraction indicated that its main components were K-feldspar and quartz. Pyroxenes (specifically ferrosilite), dolomite, calcite, plagioclase, and zincite were also present.

#### High‑intensity magnetic separation

High-intensity magnetic separation (HIMS) applied to the 0.075–4.76 mm soil fraction divided the material into a magnetic fraction accounting for 15% of the − 4.76 + 0.075 mm mass and a nonmagnetic fraction representing the remaining 85% (Table 6). Metal removal efficiencies varied considerably among elements, with antimony (83%), lead (76%), arsenic (75%), and zinc (70%) showing the highest reductions, whereas mercury (14%) and selenium (16%) exhibited comparatively limited removal.

**Table 6:** Heavy metal concentrations in the magnetic and nonmagnetic fractions of the −4.76 + 0.075 mm size range. Values indicated with < 5 represent concentrations below the element-specific limit of quantification (5 mg/kg)

	−4.76 + 0.075 mm Nonmagnetic	−4.76 + 0.075 mm Magnetic
Mass %	85.1 ± 0.6%	14.9 ± 0.6%
Specific gravity (g/cm^3^)	2.66 ± 0.02	2.92 ± 0.01
Antimony (mg/kg)	8.2 ± 1.0	260 ± 30
Arsenic (mg/kg)	21.1 ± 2.3	437 ± 48
Cadmium (mg/kg)	236 ± 22	2640 ± 243
Chromium (mg/kg)	6.0 ± 0.7	238 ± 28
Cobalt (mg/kg)	< 5	33.6 ± 2.2
Copper (mg/kg)	52.8 ± 3.9	1880 ± 139
Iron (mg/kg)	4880 ± 400	92,800 ± 7610
Lead (mg/kg)	1940 ± 223	38,800 ± 4462
Mercury (mg/kg)	30.8 ± 4.8	42.1 ± 6.6
Nickel (mg/kg)	< 5	91.8 ± 6.2
Selenium (mg/kg)	46.1 ± 3.2	72.9 ± 5.0
Thallium (mg/kg)	2.9 ± 0.2	36.7 ± 2.8
Tin (mg/kg)	< 5	139 ± 16
Titanium (mg/kg)	102 ± 18	1180 ± 207
Vanadium (mg/kg)	7.4 ± 0.8	64.8 ± 6.6
Zinc (mg/kg)	5280 ± 364	76,600 ± 5285

The magnetic fraction showed higher concentrations of contaminants compared to the original soil; specifically, antimony was almost five times more concentrated, cadmium approximately two times, and other elements about three times more concentrated. Mineralogical analysis identified the presence of predominantly diamagnetic minerals: K-feldspar, cerussite, plagioclase, gypsum, dolomite, and zincite. The only paramagnetic mineral detected was pyroxene, specifically ferrosilite.

**Fig. 6:**
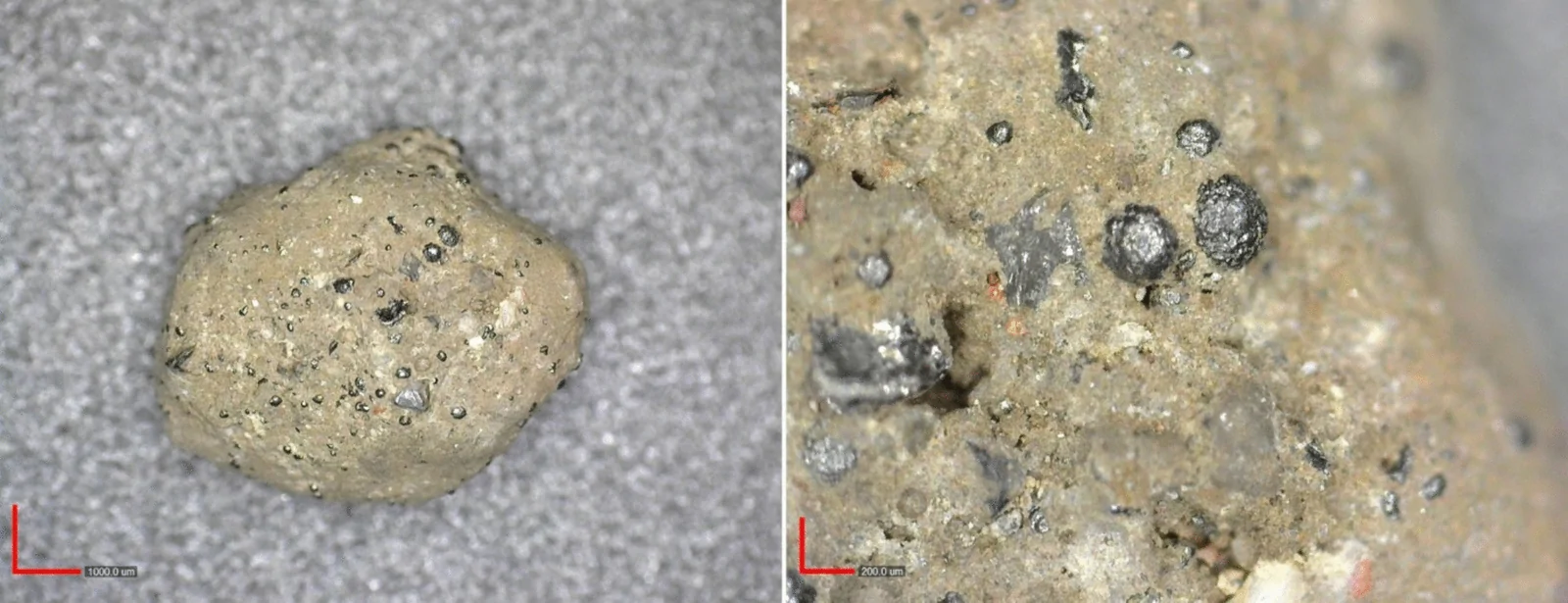
Optical microscopy images of impurities in a soil particle of the −4.76 + 0.075 mm magnetic fraction

Optical microscopy images of particles from the −4.76 + 0.075 mm magnetic fraction (Fig. 6) revealed numerous dark inclusions dispersed within the light brown mineral matrix.

Contaminant concentrations in the nonmagnetic fraction were reduced from 4 to 19 times relative to the original soil. This reduction results in lowering the amount of antimony and arsenic under the Italian soil contamination threshold levels for industrial sites (D.lgs. n. 152/2006). However, the concentrations of cadmium, lead, mercury, selenium, and zinc remained above their respective limits by factors of 16, 2, 6, 3 and 3.5.

**Fig. 7:**
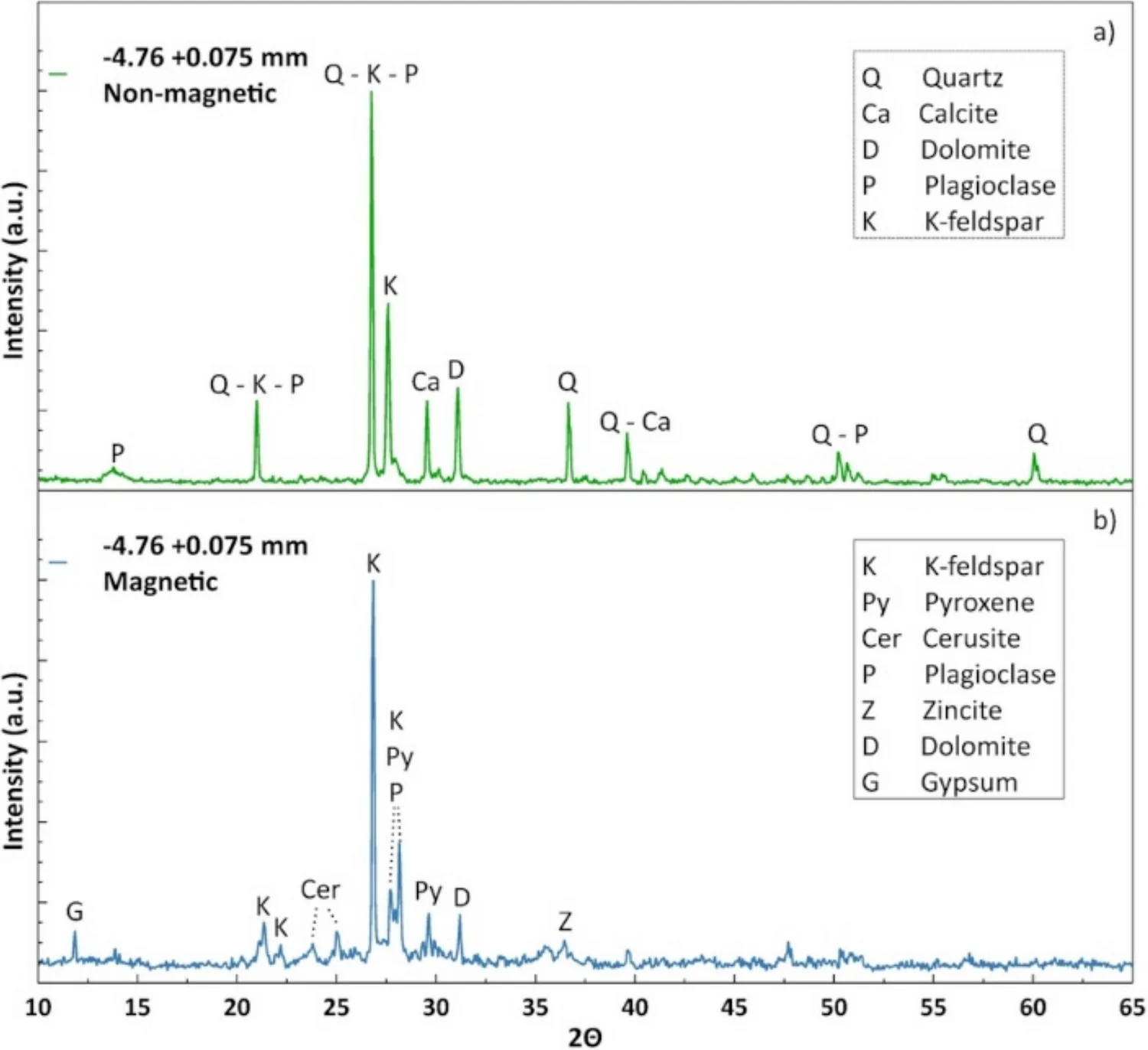
X-ray diffractogram of −4.76 + 0.075 mm after magnetic separation: **a** nonmagnetic fraction and **b** magnetic fraction

Mineralogical analysis of the −4.76 + 0.075 mm nonmagnetic fraction showed the presence of quartz (SiO_2_), calcite (CaCO_3_), dolomite (CaMg(CO_3_)_2_), plagioclase (tectosilicate with Ca, Al, Na), and K-feldspar (potassium-aluminum silicate) (Fig. 7).

### Efficiency of EDTA-assisted chemical extraction

Heavy metal concentrations measured after chemical extractions with EDTA, together with those assessed after the control test, are reported in Table 7. No significant change in metal concentrations was observed after the control test performed without EDTA.

**Table 7:** Heavy metal concentrations after EDTA-assisted chemical extractions (15–75 mM). The control represents the extraction test carried out without EDTA under the same operational conditions. Values indicated with < 5 represent concentrations below the element-specific limit of quantification (5 mg/kg)

	Control	EDTA 15 mM	EDTA 30 mM	EDTA 45 mM	EDTA 60 mM	EDTA 75 mM
Antimony (mg/kg)	8.1 ± 0.9	5.3 ± 0.6	< 5	< 5	< 5	< 5
Arsenic (mg/kg)	21.0 ± 2.3	20.9 ± 2.3	18.4 ± 2.0	16.9 ± 1.9	10.8 ± 1.2	9.5 ± 1.0
Cadmium (mg/kg)	232 ± 21	152 ± 14	77 ± 7.1	54.7 ± 5.0	51.3 ± 4.7	48.6 ± 4.5
Chromium (mg/kg)	5.9 ± 0.7	< 5	< 5	< 5	< 5	< 5
Cobalt (mg/kg)	< 5	< 5	< 5	< 5	< 5	< 5
Copper (mg/kg)	50.6 ± 3.7	40.2 ± 3.0	33.1 ± 2.4	28.2 ± 2.1	27.5 ± 2.0	26.4 ± 2.0
Iron (mg/kg)	4808 ± 394	4250 ± 349	4220 ± 346	4190 ± 344	4030 ± 331	3760 ± 308
Lead (mg/kg)	1895 ± 218	1340 ± 154	987 ± 114	930 ± 107	921 ± 106	873 ± 104
Mercury (mg/kg)	30.2 ± 4.7	19.7 ± 3.1	18.6 ± 2.9	16.3 ± 2.6	12.6 ± 2.0	12.3 ± 1.9
Nickel (mg/kg)	< 5	< 5	< 5	< 5	< 5	< 5
Selenium (mg/kg)	45.1 ± 3.1	28.7 ± 2.0	27.6 ± 1.9	24.7 ± 1.7	24.0 ± 1.7	17.8 ± 1.2
Thallium (mg/kg)	2.9 ± 0.2	2.6 ± 0.2	2.5 ± 0.2	2.4 ± 0.2	2.3 ± 0.2	2.0 ± 0.2
Tin (mg/kg)	< 5	< 5	< 5	< 5	< 5	< 5
Titanium (mg/kg)	99.1 ± 17.3	82.4 ± 14.4	80.3 ± 14	78.1 ± 13.7	77.3 ± 13.5	75.3 ± 13.2
Vanadium (mg/kg)	7.3 ± 0.7	< 5	< 5	< 5	< 5	< 5
Zinc (mg/kg)	5211 ± 360	4400 ± 304	3731 ± 257	3062 ± 211	2970 ± 205	2740 ± 189

Chemical extraction demonstrated high efficiency in reducing cadmium content, with its concentration in the −4.76 + 0.075 mm nonmagnetic fraction decreasing from 232 to 48.6 mg/kg, representing a reduction of approximately a factor of 5.

Similarly, the concentrations of mercury, selenium, arsenic, copper, lead, and zinc were also significantly reduced by a factor of 2 to 2.5. Conversely, concentrations of iron, thallium, and titanium were reduced by less than a factor of 1.5.

**Fig. 8:**
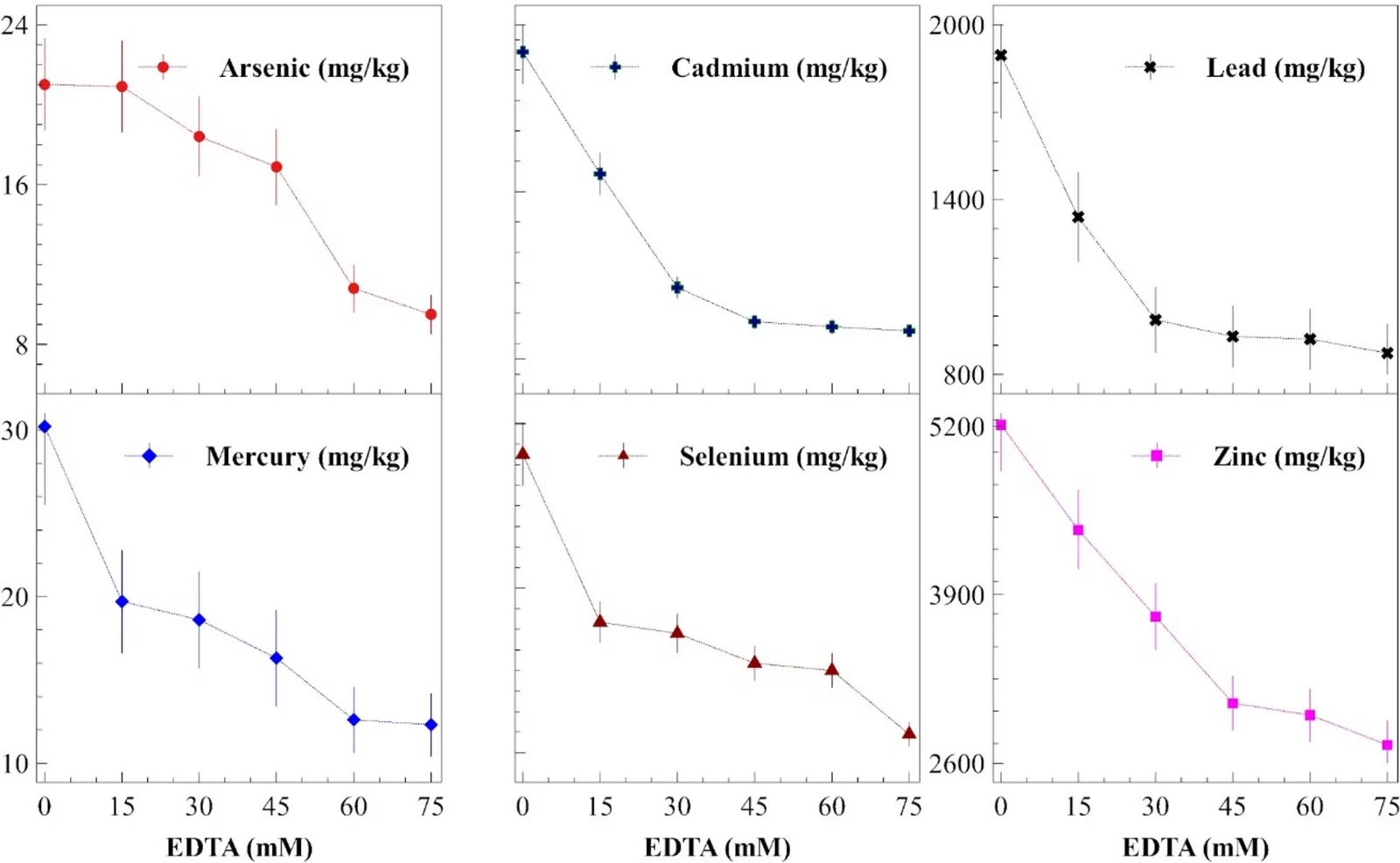
Metal concentration as a function of EDTA concentration during chemical extraction

Increasing the amount of EDTA generally led to a decrease in heavy metal concentrations (Fig. 8). Cadmium, lead, copper, and zinc exhibited a similar trend, as their contents decreased up to 45 mM EDTA, beyond which their concentrations remained relatively constant. Specifically, zinc exhibited a linear decrease up to 45 mM EDTA. For arsenic, a minimum EDTA concentration of 60 mM was necessary to achieve a twofold concentration reduction.

Iron, mercury, selenium, and titanium concentrations showed a notable decrease at 15 mM EDTA; at higher concentrations, their amounts decreased only slightly, with a final overall decrease observed only for iron and selenium.

Lead concentrations fell below Italian soil contamination threshold level for industrial sites (D.lgs. n. 152/2006) using 30 mM of EDTA, and selenium level approached its threshold with 75 mM EDTA. Cadmium, mercury, and zinc concentrations were still 3, 2.5, and 1.8 times higher than their respective thresholds.

### Human health risk assessment

#### Human health risk assessment of original soil

As mentioned above, the original soil was heavily contaminated with numerous heavy metals, posing significant human health risks through various exposure pathways.

The hazard quotients (HQs) through ingestion, dermal contact, and inhalation as well as the combined hazard indexes are associated with the exposure to the original soil for nonresidential and residential scenarios (Table 8).

**Table 8:** Summary of noncarcinogenic health risk assessment of the original soil for nonresidential (outdoor worker) and residential (adult and child) exposure scenarios. Values greater than unity are highlighted in bold

	Outdoor worker				Residential adult				Residential child			
	HQ_ing_	HQ_derm_	HQ_inh_	HI	HQ_ing_	HQ_derm_	HQ_inh_	HI	HQ_ing_	HQ_derm_	HQ_inh_	HI
**Sb**	1.4E−01	6.2E−02	9.7E−05	2.0E−01	2.3E−01	6.1E−02	1.6E−04	2.9E−01	**2.1E + 00**	4.0E−01	7.3E−04	**2.5E + 00**
**As**	4.7E−01	9.2E−02	4.8E−03	5.6E−01	7.6E−01	9.1E−02	7.8E−03	8.6E−01	**7.1E + 00**	5.9E−01	3.6E−02	**7.7E + 00**
**Cd**	**1.0E + 01**	**2.7E + 00**	5.2E−02	**1.3E + 01**	**1.6E + 01**	**2.6E + 00**	8.4E−02	**1.9E + 01**	**1.5E + 02**	**1.7E + 01**	3.9E−01	**1.7E + 02**
Cr	5.0E−05	2.5E−04	n.a	3.0E−04	8.1E−05	2.5E−04	n.a	3.3E−04	7.6E−04	1.6E−03	n.a	2.4E−03
Co	3.4E−02	2.2E−03	8.7E−04	3.7E−02	5.5E−02	2.2E−03	1.4E−03	5.9E−02	5.1E−01	1.4E−02	6.6E−03	5.4E−01
Cu	1.7E−02	1.1E−03	n.a	1.8E−02	2.7E−02	1.1E−03	n.a	2.8E−02	2.5E−01	7.1E−03	n.a	2.6E−01
Fe	3.2E−02	2.1E−03	n.a	3.4E−02	5.2E−02	2.1E−03	n.a	5.5E−02	4.9E−01	1.4E−02	n.a	5.0E−01
**Hg**	1.6E−01	1.3E−02	8.2E−05	1.7E−01	2.6E−01	1.3E−02	1.3E−04	2.7E−01	**2.4E + 00**	8.5E−02	6.2E−04	**2.5E + 00**
Ni	1.9E−03	3.2E−03	5.5E−04	5.7E−03	3.2E−03	3.1E−03	8.9E−04	7.2E−03	2.9E−02	2.1E−02	4.2E−03	5.4E−02
Se	1.1E−02	9.3E−04	1.5E−06	1.2E−02	1.8E−02	9.1E−04	2.4E−06	1.9E−02	1.7E−01	6.0E−03	1.1E−05	1.8E−01
**Tl**	1.0E + 00	6.3E−02	n.a	**1.0E + 00**	**1.6E + 00**	6.2E−02	n.a	**1.6E + 00**	**1.5E + 01**	4.1E−01	n.a	**1.5E + 01**
Sn	7.6E−05	5.0E−06	n.a	8.1E−05	1.2E−04	4.9E−06	n.a	1.3E−04	1.1E−03	3.2E−05	n.a	1.2E−03
Ti	1.1E−04	7.2E−06	n.a	1.2E−04	1.8E−04	7.1E−06	n.a	1.9E−04	1.7E−03	4.7E−05	n.a	1.7E−03
V	3.6E−03	9.1E−03	9.3E−05	1.3E−02	5.8E−03	8.9E−03	1.5E−04	1.5E−02	5.4E−02	5.8E−02	7.0E−04	1.1E−01
**Zn**	8.7E−02	5.7E−03	n.a	9.2E−02	1.4E−01	5.6E−03	n.a	1.5E−01	**1.3E + 00**	3.7E−02	n.a	**1.3E + 00**

In both nonresidential and residential scenarios, the average daily intakes through inhalation were lower than the inhalation reference dose (HQ_inh_ < 1) for all heavy metals.

For *the nonresidential outdoor worker scenario*, the ingestion (HQ_ing_) and dermal contact (HQ_derm_) hazard quotients, as well as the hazard indexes (HI), were below 1 for all heavy metals except for cadmium and thallium. For cadmium, HQ_ing_ was 10.0, HQ_derm_ was 2.7, and HI was 12.7. Thallium also exceeded the threshold, with an HQ_ing_ of 1 and an HI of 1.1, while its HQ_derm_ was below 1.

Similarly in the *residential adult* scenario the HQ_ing_ and HQ_derm_ and hazard indexes for all heavy metals were below 1, except for cadmium and thallium. Cadmium showed the highest values, with an HQ_ing_ of 16.3, HQ_derm_ of 2.6, and an HI of 19.0, while thallium slightly exceeded unity, with an HQ_ing_ of 1.6 and an HI of 1.6.

In *the residential child scenario*, the potential health risk for children is significant, particularly due to the concentrations of cadmium, thallium, arsenic, antimony, mercury, and zinc. The ingestion hazard quotients (HQ_ing_) and hazard indexes (HI) for these metals all exceeded unity (HQ_ing_ > 1, HI > 1). Zinc, antimony, and mercury pose a minor noncarcinogenic health risk, with HQ_ing_ and HI values ranging from 1.3 to 2.5. For arsenic, HQ_ing_ value was 7.1 and HI was 7.7.

The concentrations of cadmium and thallium in the original soil exhibited the most significant noncarcinogenic risk. For cadmium, HQ_ing_ was 152.0 and HQ_derm_ was 17.0, resulting in a total HI of 169.4. For thallium, HQ_ing_ was 14.5 and HI was 14.9 while HQ_derm_ was less than 1.

*Carcinogenic risks* for the potential carcinogens arsenic, cadmium, and nickel are reported in Table 9.

**Table 9:** Carcinogenic risk of arsenic (As), cadmium (Cd), and nickel (Ni) for nonresidential (outdoor worker) and residential (adult and child) exposure scenarios. Values greater than the US Environmental Protection Agency (USEPA) threshold of 10^–4^ are highlighted in bold. For elements with concentrations lower than the limit of quantification, the cancerogenic risks were not estimated

Carcinogenic risk		Original soil	Physical separation		EDTA				
			−4.76 + 0.075 mm	Nonmagnetic	15 mM	30 mM	45 mM	60 mM	75 mM
Outdoor worker	As CR_ing_	7.5E−05	3.9E−05	1.0E−05	9.9E−06	8.7E−06	8.0E−06	5.1E−06	4.5E−06
	As CR_derm_	1.5E−05	7.8E−06	2.0E−06	2.0E−06	1.7E−06	1.6E−06	1.0E−06	8.9E−07
	As CR_inh_	3.2E−08	1.7E−11	4.2E−12	4.2E−12	3.7E−12	3.4E−12	2.1E−12	1.9E−12
	As CR	9.0E−05	4.7E−05	1.2E−05	1.2E−05	1.0E−05	9.5E−06	6.1E−06	5.4E−06
	Cd CR_inh_	9.5E−08	4.9E−11	2.0E−11	1.3E−11	6.4E−12	4.6E−12	4.3E−12	4.0E−12
	Ni CR_inh_	2.6E−09	2.3E−09	-	-	-	-	-	-
Residential adult	As CR_ing_	**1.5E−04**	7.7E−05	1.9E−05	1.9E−05	1.7E−05	1.6E−05	9.9E−06	8.7E−06
	As CR_derm_	1.7E−05	9.2E−06	2.3E−06	2.3E−06	2.0E−06	1.9E−06	1.2E−06	1.0E−06
	As CR_inh_	6.2E−08	3.2E−11	8.2E−12	8.1E−12	7.1E−12	6.5E−12	4.2E−12	3.7E−12
	As CR	**1.6E−04**	8.6E−05	2.2E−05	2.1E−05	1.9E−05	1.7E−05	1.1E−05	9.8E−06
	Cd CR_inh_	1.8E−07	9.5E−11	3.8E−11	2.5E−11	1.2E−11	8.9E−12	8.3E−12	7.9E−12
	Ni CR_inh_	4.3E−09	3.7E−09	-	-	-	-	-	-
Residential child	As CR_ing_	**2.7E−04**	**1.4E−04**	3.6E−05	3.6E−05	3.2E−05	2.9E−05	1.9E−05	1.6E−05
	As CR_derm_	4.7E−05	2.5E−05	6.2E−06	6.1E−06	5.4E−06	4.9E−06	3.2E−06	2.8E−06
	As CR_inh_	5.7E−08	3.0E−11	7.6E−12	7.6E−12	6.6E−12	6.1E−12	3.9E−12	3.4E−12
	As CR	**3.2E−04**	**1.7E−04**	4.2E−05	4.2E−05	3.7E−05	3.4E−05	2.2E−05	1.9E−05
	Cd CR_inh_	1.7E−07	8.8E−11	3.6E−11	2.3E−11	1.2E−11	8.3E−12	7.8E−12	7.4E−12
	Ni CR_inh_	2.0E−08	1.7E−08	-	-	-	-	-	-

The carcinogenic risk of the original soil associated with *cadmium* and *nickel* was negligible in both residential and nonresidential scenarios, with carcinogenic risk (CR) values ranging from 2.6 × 10^–9^ to 1.8 × 10^–7^. Similarly, risk from arsenic exposure via inhalation was also negligible (CR_inh_ < 3.2 × 10^−8^).

For the nonresidential outdoor worker scenario, the carcinogenic risk from *arsenic* is within the tolerable range, with a CR value between 1.5 × 10^–5^ and 7.5 × 10^–5^.

In the residential scenario, the total and ingestion-related carcinogenic risk of arsenic exceeded the USEPA threshold of 10^–4^ (USEPA 1991; Fowle and Dearfield 2000) for both adults and children, with children presenting the highest vulnerability.

Lead-related risk estimated as PbB_fetal,0.95_ was 51 µg/dL (Table 10), five times higher than the established reference value of 10 µg/dL for fetal blood lead concentrations (USEPA 2003).

**Table 10:** Assessment of risk associated with lead exposure: blood lead concentrations for exposed adults (PbB_adult,central_) and fetal blood lead concentration (PbB_fetal,0.95_) for original soil and after physical and chemical remediation treatment

	Original soil	Physical separation		Chemical extraction with EDTA				
		−4.76 + 0.075 mm	Nonmagnetic	15 mM	30 mM	45 mM	45 mM	75 mM
PbB_adult,central_ (μg/dL)	21.6	13.5	4.5	3.6	3.1	3.0	3.0	3.0
PbB_fetal,0.95_ (μg/dL)	51.1	31.9	10.6	8.6	7.4	7.2	7.2	7.0

#### Human health risk assessment of soil after physical separation

Mechanical screening provided an initial risk reduction, achieving removal efficiencies ranging from 14% (nickel) to 53% (copper) (Table 11).

**Table 11:** Heavy metal removal efficiencies (%) for mechanical screening, the combined mechanical screening and magnetic separation, and the chemical extraction using EDTA concentrations from 15 to 75 mM. For elements with concentrations lower than the limit of quantification, the removal efficiencies were not estimated

Element	Physical separation		Chemical extraction				
	Mechanical screening	Mechanical and magnetic separation	EDTA 15 mM	EDTA 30 mM	EDTA 45 mM	EDTA 60 mM	EDTA 75 mM
Antimony	27%	87%	92%	-	-	-	-
Arsenic	47%	87%	87%	88%	89%	93%	94%
Cadmium	49%	79%	87%	93%	95%	96%	96%
Chromium	47%	93%	-	-	-	-	-
Cobalt	21%	-	-	-	-	-	-
Copper	53%	93%	95%	96%	96%	96%	97%
Iron	23%	81%	83%	84%	84%	84%	85%
Lead	41%	86%	90%	93%	93%	93%	94%
Mercury	35%	44%	64%	66%	70%	77%	77%
Nickel	14%	-	-	-	-	-	-
Selenium	14%	28%	55%	57%	61%	63%	72%
Thallium	23%	73%	77%	78%	78%	79%	82%
Tin	53%	-	-	-	-	-	-
Titanium	26%	73%	78%	79%	79%	79%	80%
Vanadium	17%	64%	-	-	-	-	-
Zinc	41%	82%	85%	87%	90%	90%	91%

**Fig. 9:**
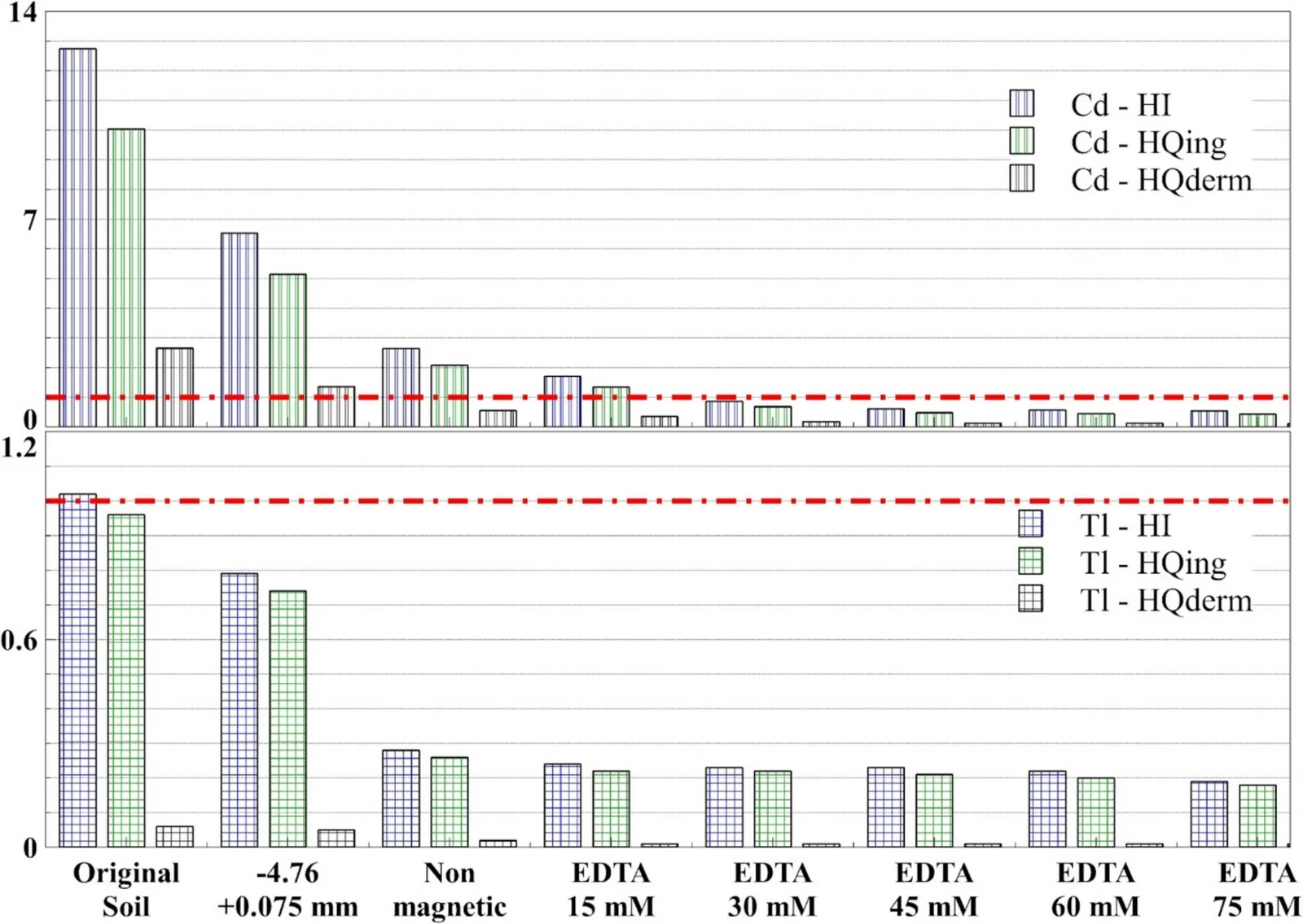
Noncarcinogenic health risk assessment for nonresidential outdoor worker scenario: hazard quotients and hazard indexes of cadmium (Cd) and thallium (Tl). The red line indicates the USEPA threshold for noncarcinogenic risk

**Fig. 10:**
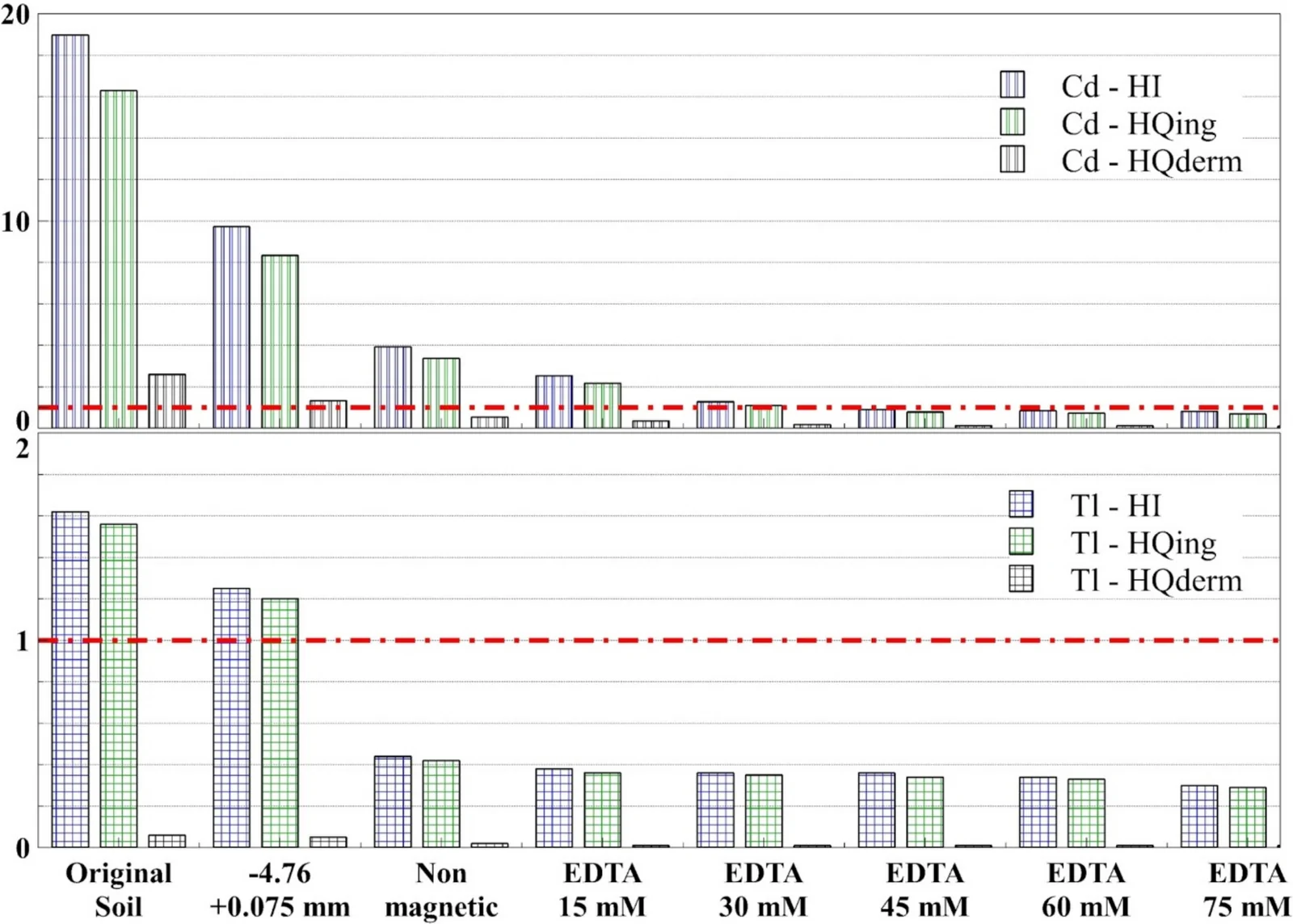
Noncarcinogenic health risk assessment for residential adult scenario: hazard quotients and hazard indexes of cadmium (Cd) and thallium (Tl). The red line indicates the USEPA threshold for noncarcinogenic risk

**Fig. 11:**
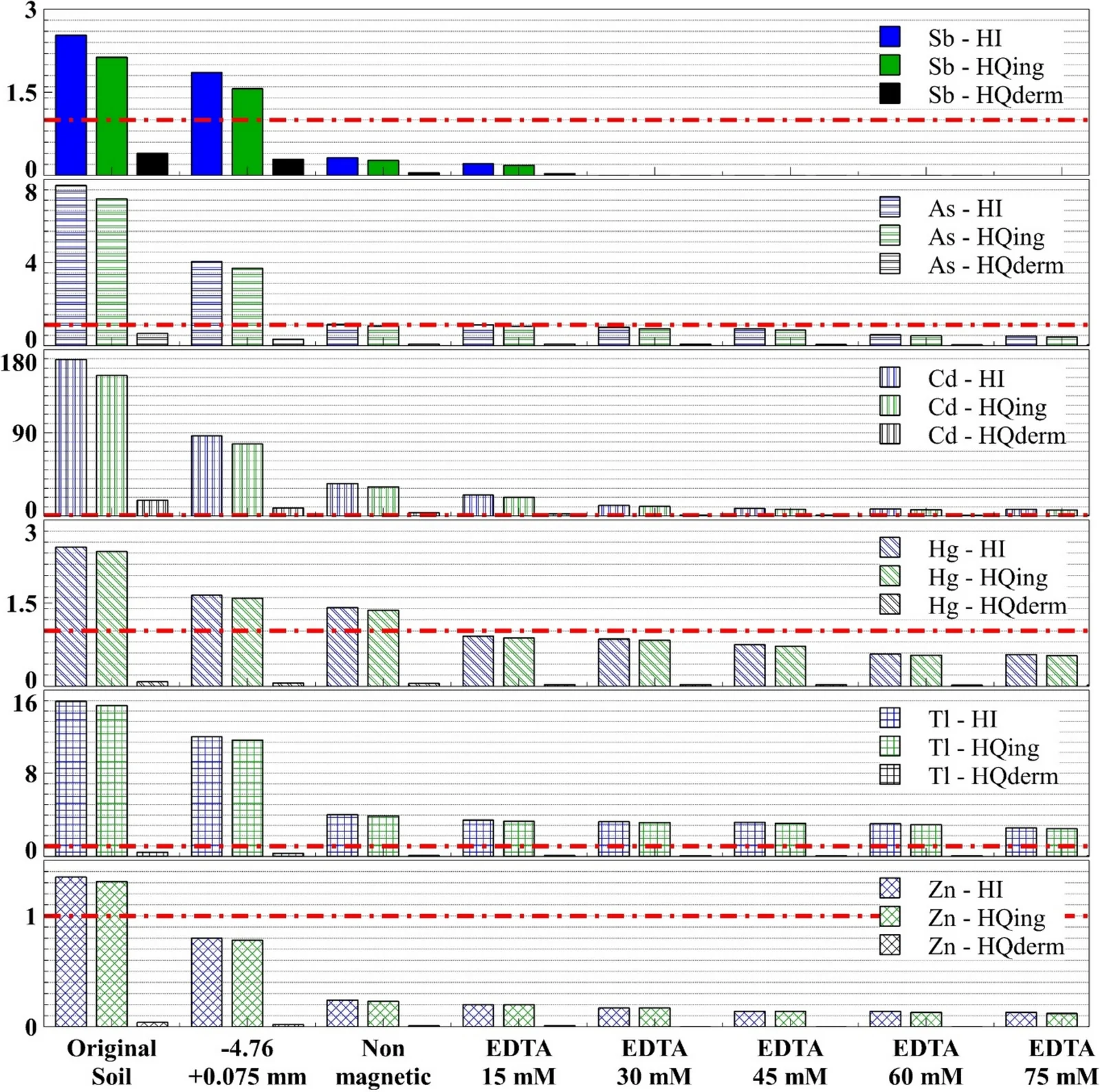
Noncarcinogenic health risk assessment for residential child scenario: hazard quotients and hazard indexes of antimony (Sb), arsenic (As), cadmium (Cd), mercury (Hg), nickel (Ni), thallium (Tl), and zinc (Zn). The red line indicates the USEPA threshold for noncarcinogenic risk

Although the cadmium concentration was halved, HQ_ing_, HQ_derm_, and HI values still exceeded unity in both nonresidential and residential scenarios (Figs. 9, 10, and 11).

A 23% reduction in thallium concentration resulted in HQ_ing_ and HI values falling below 1 for outdoor workers (Fig. 9), though HQ_ing_ remained above unity for residential adults (Fig. 10). For children, however, the risk associated with thallium remains high, with HQ_ing_ and HI values of 11.2 and 11.5, respectively (Fig. 11).

The 41% reduction in zinc concentration was sufficient to eliminate the associated noncarcinogenic health risk for children (HQ_ing_ < 1 and HI < 1). While the concentration of antimony and mercury still posed a minor risk, the noncarcinogenic health risk from arsenic remained significant despite its concentration being halved (Fig. 11).

In addition, the carcinogenic risk (Table 9) was within the tolerable range for adults, but for children, the total and ingestion-related carcinogenic risk of arsenic exceeded the USEPA threshold of 10^−4^ (USEPA 1991; Fowle and Dearfield 2000).

The magnetic separation step provided a more significant reduction in risks due to a substantial decrease in most contaminant concentrations (Table 11), with most being reduced by 73 to 93%. The exceptions were mercury, selenium, and vanadium, which experienced reductions of 44%, 28%, and 64%, respectively. Notably, the concentrations of cobalt, nickel, and tin fell below the limit of quantification.

For residential adults, HQ_ing_ and HI of thallium fall below 1 (Fig. 10). For children (Fig. 11), the noncarcinogenic health risk associated with arsenic and antimony became negligible. However, HQ_ing_, HQ_derm_, and HI for cadmium continued to exceed unity, and the concentrations of mercury and thallium pose a minor noncarcinogenic health risk for children.

Furthermore, the carcinogenic risks associated with arsenic reached a negligible or tolerable level for children as well (Table 9). The fetal blood lead concentration was reduced to 11 µg/dL (Table 10), which is only slightly higher than the goal of 10 µg/dL.

#### Human health risk assessment after chemical extraction

The chemical extraction step significantly improved the overall removal efficiency (Table 11). The overall efficiency ranged from 72% (selenium) to 97% (copper). This process further reduced concentrations of mercury by 33%, selenium by 44%, and cadmium by 17%, beyond the reductions achieved by physical separation. For arsenic, lead, thallium, titanium, and zinc, this additional reduction ranged between 7 and 9%, while for iron and copper, it was negligible (4%). Notably, using 30 mM EDTA, the concentrations of antimony fell below the limit of quantification.

This step proved critical in lowering health risks (Figs. 9, 10, and 11). For cadmium, HQ_ing_ and HI were successfully reduced to acceptable values (below 1), following extraction with 30 mM EDTA for the outdoor worker scenario and 45 mM EDTA for the residential adult scenario.

*For the residential children scenario* (Fig. 11), the noncarcinogenic health risk associated with mercury exposure was reduced to acceptable levels after chemical extraction with 15 mM EDTA (HQ_ing_ < 1 and HI < 1). Although physical separation and chemical extraction lowered the concentrations of cadmium and thallium, they were insufficient to reduce the ingestion hazard quotients and the hazard index for children below 1. Specifically, for cadmium, the HQ_ing_ and HI values remained at 6.5 and 7.2, respectively, while for thallium both HQ_ing_ and HI remained at 2.7. However, the HQ_derm_ value for cadmium fell below 1 after chemical extraction with 45 mM EDTA (Fig. 11).

Finally, the PbB_fetal,0.95_ goal was reached (Table 10), specifically after treatment with 15 mM EDTA.

Following physical separation and chemical extraction with 45 mM EDTA, the remediated soil posed no significant human health risk for outdoor workers and residential adults. This was because neither hazard quotients nor hazard indexes exceeded unity, and carcinogenic risks fell within acceptable ranges. Additionally, the fetal blood lead concentration (Table 10) also reached the established reference value.

However, a significant noncarcinogenic health risk remains for children due to cadmium and thallium concentrations.

## Discussion

### Multimetal contamination patterns and implications for remediation

The original soil exhibited severe multimetal contamination (Table 4). The extremely elevated concentrations of iron (25,695 mg/kg), lead (13,800 mg/kg), and zinc (29,500 mg/kg) indicate that the soil’s metal sorption capacity has been exceeded, leading to the presence of contaminants as discrete metal-bearing mineral phases rather than adsorbed ions on soil particles (Peters 1999). This interpretation is supported by the X-ray diffraction results, which identified zincite (ZnO), cerussite (PbCO_3_), ferrosilite (Fe_2_Si_2_O_6_), and additional iron-bearing phases (Fig. 2). Cadmium, mercury copper, antimony, arsenic, and selenium were also detected at high concentrations. These elements are common impurities in both zinc and lead ores (Sinclair 2005; Dunne et al. 2019), reflecting the site’s history of zinc and lead production and underscoring the need for a comprehensive remediation strategy.

Ferrosilite (Fe_2_Si_2_O6) and other iron mineral phases could act as carrier phases or scavengers for other pollutants (Rikers et al. 1998; Ettler 2016). For example, arsenic could form inner-sphere complexes that are difficult to remove without dissolving the iron host (Liao et al. 2016). Because many of these Fe-bearing particles exhibit paramagnetic behavior, high-intensity magnetic separation (HIMS) could be a viable treatment step to reduce the volume of contaminated material by concentrating these metal-rich particles (Rikers et al. 1998; Dermont et al. 2008; Zhang et al. 2025b).

The distribution of heavy metals across the different particle size fractions showed that the finest fractions (−0.010 mm and + 0.010–0.075 mm) exhibited the highest contamination levels. The preferential accumulation of metals in the fine fractions (< 0.075 mm) reflects the well-established principle that heavy metals and metalloids tend to concentrate in the fine soil fraction (silt and clay) due to their larger specific surface areas and high reactivity of fine particles, which provide more sites for the adsorption and binding of metals like Pb, Cd, Zn, and As (Peters 1999; Dermont et al. 2008; Liao et al. 2016; Zhang et al. 2022).

Based on these results, the material smaller than 0.075 mm, identified as the most heavily contaminated, was designated for disposal.

Given the mineralogical complexity and high concentrations, a single remediation method may not be effective (Reddy and Chinthamreddy 2000). An integrated approach, combining physical separation, to recover cleaner coarse fractions, and chemical extraction, to solubilize the residual contaminants from soil particles, is considered the most robust approach for permanent soil restoration in industrial contexts (Reddy and Chinthamreddy 2000; Dermont et al. 2008). However, aggressive chemical extraction can compromise soil structure and fertility, so remediation strategies must balance high contaminant-removal efficiency with the preservation of soil functionality to ensure successful revegetation or future industrial reuse (Guo et al. 2018; Wang et al. 2025).

### Effectiveness of physical separation process

The *physical separation steps* (mechanical screening and magnetic separation) resulted in substantial metal concentration and volume reduction, consistent with previous studies reporting enrichment factors of 2–10 for various metals in fine soil fractions (Ko et al. 2006; Dermont et al. 2008; Zheng et al. 2022).

By separating the soil at the 0.075-mm threshold, 79% of the total sample mass was recovered as a cleaner coarse fraction. This agrees with the literature stating that mechanical separation is most cost-effective and applicable when the sand and gravel content exceeds 50–70%, as seen in this sample, where the coarse fraction represents nearly 80% (Ko et al. 2006; Dermont et al. 2008; Zhang et al. 2022). The fact that this fraction is 1.5 to 2 times less contaminated than the original soil suggests it could potentially be suitable for reuse or backfill after minimal additional treatment (Ko et al. 2006; Zheng et al. 2022).

*High-intensity magnetic separation* (HIMS) applied to the 0.075–4.76 mm soil fraction provided complementary metal removal. The process successfully isolated 85% of the soil mass as a nonmagnetic fraction, whereas the remaining 15%, consisting of a highly contaminated magnetic concentrate (Table 6), was designated for disposal.

The separation showed high selectivity, with antimony (83%), lead (76%), arsenic (75%), and zinc (70%) achieving the greatest reductions (Table 11). These metals and metalloids are often associated with iron-bearing minerals in smelting environments (Rikers et al. 1998). Conversely, the limited removal of mercury (14%) and selenium (16%) suggests these elements are not strongly associated with the magnetic phases and may instead be bound to discrete forms less susceptible to magnetic fields (Mulligan et al. 2001).

Although the mineralogical analysis of the magnetic fraction identified diamagnetic minerals such as cerussite, zincite, gypsum, and dolomite (Dunne et al. 2019), their recovery through high‑intensity magnetic separation is attributable to an indirect mechanism. This process is possible because diamagnetic heavy metal minerals are frequently associated with paramagnetic impurities, such as iron-bearing minerals or amorphous iron oxides, which act as carrier phases and impart sufficient magnetic susceptibility to enable particle capture under a strong magnetic field. This mechanism is consistent with the behavior of heavy metals such as Pb and Zn, which typically form diamagnetic mineral species associated with iron‑rich phases in contaminated industrial soils. (Rikers et al. 1998; Mercier et al. 2001; Ibrahim et al. 2017; Tripathy et al. 2017). Further evidence supporting this interpretation is provided by the optical microscopy images of the − 4.76 + 0.075 mm magnetic fraction, which clearly reveal dark, most likely iron-rich inclusions embedded within the light brown diamagnetic mineral matrix (Fig. 6).

Considering the concentration value of iron, zinc, and lead in the nonmagnetic fraction (Table 6), together with the absence of diffraction peaks corresponding to their respective metal-bearing mineral phases (Fig. 7), the results indicate that, after physical separation, these contaminants were present solely in an adsorbed form on the soil particles (Peters 1999). This interpretation is supported by the difference in density between the magnetic and nonmagnetic fractions, which is consistent with the removal of mineral-bound metals during high-intensity magnetic separation.

These results prove that magnetic separation is a valuable complement to size-based separation (Liao et al. 2016). It prepares the soil for a more targeted second stage of remediation, such as chelant extraction (EDTA), which can effectively remove the remaining adsorbed fractions from the nonmagnetic soil (Griffiths 1995; Peters 1999; Dermont et al. 2008).

### Chemical extraction performance

Chemical extraction with EDTA proved highly effective in mobilizing several heavy metals from the − 4.76 + 0.075 mm nonmagnetic fraction (Tables 6 and 7). The control test performed without EDTA did not result in any appreciable variation in metal concentrations, confirming that the reductions observed during the chemical extraction step were driven by the chelating action of EDTA rather than by the extraction procedure itself. Among the analyzed metals, cadmium exhibited the highest removal efficiency, corresponding to an approximately fivefold decrease relative to its concentration after magnetic separation.

As reported in literature (Dermont et al. 2008; Zhang et al. 2022), EDTA is highly effective at removing cadmium due to its strong ability to form stable, water-soluble complexes with this metal. The simultaneous reduction of mercury, selenium, arsenic, copper, lead, and zinc by approximately 2–2.5-fold confirms EDTA’s role as a broad‑spectrum extractant capable of addressing multimetal contamination typically associated with smelting sites.

Increasing EDTA concentration enhanced metal removal up to 45 mM for Cd, Pb, Cu, and Zn, after which their concentrations plateaued (Fig. 8). The primary target for EDTA includes metals in water-soluble, exchangeable, and carbonate-bound forms and the extraction from these fractions is typically rapid. Beyond the 45 mM threshold, these pools become depleted, and further additions of the chelating agent provide negligible benefit because the remaining metals are no longer accessible (Cline and Reed 1995; Peters 1999; Dermont et al. 2008; Feng et al. 2020).

Arsenic required 60 mM EDTA concentration to achieve a twofold reduction, likely because it predominantly exists as an oxyanion (arsenate or arsenite) for which EDTA exhibits low affinity. Its removal occurs by indirect dissolution of iron oxide–carrying phases, a process that requires higher reagent dosages (Oh et al. 2015). The low extraction efficiency for iron (Fe) compared to toxic metals like lead or cadmium can be considered an advantage for preserving the soil’s physical structure during remediation (Oh et al. 2015).

Lead fell below Italian soil contamination threshold levels for industrial sites (D.lgs. n. 152/2006) at 30 mM EDTA, and selenium approached its threshold at 75 mM. Despite the high technical efficiency of the process, cadmium, mercury, and zinc remained 3, 2.5, and 1.8 times above regulatory limits (D.lgs. n. 152/2006), respectively, indicating that although EDTA mobilized a significant mass of contaminants, the extreme initial metal concentrations limit the effectiveness of a single-step or single-reagent treatment (Guo et al. 2018). To achieve full compliance with the regulatory framework for the remaining Cd, Hg, and Zn, additional treatment steps—such as sequential extractions or the use of combined reagents—would likely be required to further destabilize the residual metal fractions. However, implementing such multistep or multireagent schemes at scale introduces substantial technical and economic challenges.

Although several alternatives to EDTA are available, each presents technical or economic limitations. Strong inorganic acids and alkalis can remove metals efficiently but severely compromise soil structure, deplete essential nutrients, and generate hazardous residues requiring costly neutralization and post-treatment (Dermont et al. 2008). Biodegradable chelators such as GLDA and EDDS offer improved environmental compatibility, but they are substantially more expensive and often less effective in field conditions (Zhang et al. 2022). Organic acids typically show limited extraction capacity and may induce secondary precipitation, reducing their overall efficiency (Gu et al. 2022). Biosurfactants face similar constraints: high production costs and variable affinities for different metals often necessitate multiple agents to address complex contamination mixtures (Gu et al. 2022). Biological alternatives such as phytoremediation or bioremediation are environmentally friendly, but they are extremely time-consuming, sensitive to environmental conditions, and generate metal-laden biomass that requires safe handling and disposal (Liu et al. 2018).

Collectively, these limitations indicate that extending the treatment beyond EDTA-assisted chemical extraction, whether through additional extraction cycles or complementary reagents, may drastically increase operational complexity and costs, thereby hindering the feasibility of upscaling to full-site remediation.

### Risk-based evaluation of the integrated remediation strategy

As previously mentioned, *the original soil* exhibited extensive contamination by multiple heavy metals (Table 4). These heavy metals and metalloids are persistent, nonbiodegradable, and toxic even at low concentrations, underscoring the need for comprehensive risk assessments of noncarcinogenic and carcinogenic health risks (Hashim et al. 2011; Zhang et al. 2022; Baragaño et al. 2023; Rahman et al. 2023).

The negligible risk associated with inhalation pathway in all scenario (HQ_inh_ < 1) (USEPA 2009) is consistent with previous studies indicating that, although dust resuspension may occur, direct ingestion and dermal contact generally represent the dominant exposure routes driving soil-related toxicity (Hashim et al. 2011; Ettler 2016).

Considering noncarcinogenic risks for adults, in both the nonresidential outdoor worker and residential adult scenarios, cadmium and thallium are the primary contaminants of concern. The high HI for cadmium in adults (19.0) compared with workers (12.7) is attributed to the longer exposure duration (ED) typically assumed for residents (30 years) versus workers (USEPA 1991, 2002). Cadmium is known to accumulate in the human body, leading to severe poisoning and kidney damage (Hashim et al. 2011; Oh et al. 2015; Liu et al. 2022).

The results for the residential child scenario are the most alarming, with a total cadmium HI of 169 and significant risks from Tl, As, Sb, Hg, and Zn. Children are extremely affected due to frequent hand-to-mouth activities and higher gastrointestinal absorption rates. Indeed, risk calculation parameters for children utilize a higher ingestion rate (200 mg/day vs. 100 mg/day for adults) and a substantially lower body weight (15 kg vs. 70 kg), which drastically amplifies their calculated dose (USEPA 1989, 2011).

In addition to noncarcinogenic effects, *carcinogenic risks* were also evaluated. While the carcinogenic risk for cadmium and nickel was negligible, arsenic is the most critical driver of carcinogenic risk at the site. In residential scenarios, the CR for arsenic exceeded the USEPA threshold of 10^−4^ (Fowle and Dearfield 2000), identifying it as the principal contributor to the soil’s carcinogenic potential. Arsenic is a class A carcinogen linked to skin, lung, and bladder cancers; due to its high slope factor, it poses unacceptable lifetime risks even at moderate concentrations (Oh et al. 2015; Cheraghi et al. 2025).

The lead-related risk, specifically the fetal blood lead concentration (PbB_fetal,0.95_) of 51 μg/dL, is extremely critical, as it is five times higher than established safety target (USEPA 2003). This level of exposure poses a significant risk of irreversible neurodevelopmental damage, including lower intelligence (IQ), speech and hearing issues, and behavioral disorders (USEPA 2011; Chen and Ding 2025).

*Mechanical screening* represents a valuable preliminary step for metal concentration and volume reduction; however, it is insufficient as a standalone remediation strategy to eliminate human health risks at this heavily contaminated site. Despite notable decreases in contaminant concentrations, the hazard index (HI) for several metals remained above the acceptable threshold (HI > 1), confirming the limited efficacy of this treatment phase.

Cadmium concentration was halved, but risks persisted across all exposure scenarios. This outcome is consistent with the baseline assessment, which indicated an extremely elevated HI for children (169). A reduction of this magnitude therefore still yields an HI substantially above unity, indicating that cadmium’s toxicity and its presence in soil necessitate multistep remediation strategies. A 23% reduction in thallium concentration was sufficient to reduce HI for outdoor workers below 1, but risks remained unacceptable for residents. Children, in particular, continued to experience severe noncarcinogenic risk levels (HI = 11.5). As previously discussed, this disparity is driven by the different intake parameters, resulting in a higher dose relative to adults (USEPA 1989). Arsenic continues to drive carcinogenic risk, as halving its concentration failed to reduce the CR value for children below the USEPA threshold of 10^−4^ (Fowle and Dearfield 2000), because, as reported above, even low residual concentrations are sufficient to generate unacceptable lifetime cancer risks due to its high slope factor (Cheraghi et al. 2025).

The subsequent *magnetic separation* step was able to achieve significant reductions in contaminant concentrations (73 to 93%) and associated human health risks. This enhanced performance reflects the fact that many toxic heavy metals, such as lead, zinc, arsenic, and antimony, are commonly associated with ferromagnetic or paramagnetic iron oxides (Rikers et al. 1998; Dermont et al. 2008; Baragaño et al. 2023). Thanks to this phenomenon, important remediation milestones were achieved in most sensitive population (children): The noncarcinogenic risks associated with arsenic and antimony became negligible for children, the carcinogenic risk for children associated with arsenic fell within the tolerable, and the fetal blood lead concentration was reduced to 11 µg/dL, close to the USEPA target value of 10 µg/dL (USEPA 2003).

Although the combination of mechanical and magnetic separation achieved these health-risk reductions, it did not fully eliminate them for the most vulnerable receptor group (children), particularly for cadmium (Hi = 35), thallium (Hi = 4), and mercury (HI = 1.4). To address the remaining HI > 1, chemical extraction may be required. EDTA is capable of solubilizing metals adsorbed to soil particles, potentially enabling attainment of residential safety criteria (Zhang et al. 2022; Zheng et al. 2022; Ganesh et al. 2026).

The *chemical extraction* phase demonstrates that while EDTA effectively solubilizes residual metals not removed through physical separation, the remaining risk for children continues to represent a substantial barrier to residential redevelopment.

A key outcome is that treatment with 45 mM EDTA rendered the soil safe for outdoor workers and residential adults but did not meet safety thresholds for residential children, who remained at risk from cadmium (HI = 7.2) and thallium (HI = 2.7).

Achieving the PbB_fetal,0.95_ target at a relatively low EDTA concentration (15 mM) represents a significant success. Reaching the established reference value of 10 µg/dL is critical, as fetal lead exposure is irreversibly linked to impaired neurodevelopment and lower intelligence (IQ).

Although the integrated treatment approach substantially reduced contaminant concentrations to levels acceptable for adults, persistent risks for children remain. From a management perspective, these findings indicate that without complementary management or treatment measures the remediated soil does not meet the stringent criteria required for residential land use. Nevertheless, the achieved risk reductions are consistent with the exposure assumptions and regulatory requirements of industrial or commercial land use scenarios. Thus, while the treatment demonstrates clear potential for risk mitigation, its applicability remains context-dependent and may require integration with additional remediation technologies to ensure protection of the most vulnerable population. Future in-depth studies could envisage bioaccessibility assays (e.g., PBET, SBRC), particularly in view of a potential scale-up of the remediation process, to further refine the estimation of the bioavailable fraction and the associated exposure.

## Conclusions

The original soil sample was found to be heavily contaminated with a wide variety of heavy metals, including zinc, lead, cadmium, mercury, copper, antimony, arsenic, and selenium. Specifically, several elements (cadmium, mercury, lead, zinc, arsenic, and selenium) exceeded Italian soil contamination thresholds for industrial sites by significant factors. Based on these findings, the human health risk assessment using USEPA methodologies revealed significant risks across both nonresidential and residential scenarios, with children being the most affected. In addition to the significant noncarcinogenic health risks posed by cadmium and thallium concentrations across all scenarios, arsenic, antimony, mercury, and zinc contributed to increasing the risks for children. Besides, carcinogenic risk from arsenic ingestion was found for both residential adults and children, and a further concern was the elevated risk associated with lead exposure, as the fetal blood lead concentration was five times higher than the established reference value.

Mechanical screening reduced the concentration of most heavy metals, but some health risks persisted. Cadmium and thallium continued to pose noncarcinogenic risks for both residential and nonresidential scenarios. Furthermore, children faced additional noncancer risks from arsenic, antimony, and mercury, as well as a carcinogenic risk from arsenic ingestion. Elevated fetal blood lead concentration also remained a concern at 32 µg/dL. Magnetic separation proved to be effective in significantly reducing risks, decreasing most contaminant concentrations by 73 to 93%. As a result, noncarcinogenic risks associated with arsenic and antimony and the carcinogenic risk associated with arsenic ingestion became negligible for children. Additionally, the fetal blood lead concentration dropped to 11 µg/dL, close to the target value. Physical separation alone was found to be insufficient to achieve full remediation, since cadmium, lead, mercury, selenium, thallium, and zinc still exceeded Italian soil contamination thresholds for industrial sites.

The subsequent chemical extraction step using EDTA significantly improved overall removal efficiency. Even after treatment with the highest concentration of EDTA (75 mM), cadmium, mercury, selenium, and zinc still exceeded Italian soil contamination thresholds for industrial sites. However, overall health risks were substantially reduced, particularly for outdoor workers (with 30 mM EDTA) and residential adults (with 45 mM EDTA). Treatment with 15 mM EDTA reduced the mercury-related risk for children to acceptable levels and successfully achieved the fetal blood lead concentration goal of 10 µg/dL. Despite significant reductions, noncarcinogenic risks associated with cadmium and thallium remained above acceptable levels for children.

The application of a comprehensive human health risk assessment was essential for evaluating remediation performance in terms of exposure reduction rather than concentration decrease alone. This methodology highlighted that, while the treated soil does not meet the stringent requirements for residential land use without additional remediation or management measures, the achieved risk level is adequate for commercial and industrial land use scenarios.

## Supplementary information

Below is the link to the electronic supplementary material.

ESM 1 (DOCX 76.5 KB)

## Data Availability

The data that support the findings of this study are available from the corresponding author upon reasonable request.

## Declarations

### Clinical trial number

This is not applicable.
