# Supplementary material for: Integrated physical-chemical remediation and human health risk assessment of multi-metal contaminated industrial soils
Source: Environ Sci Pollut Res Int. 2026 Sep 2;33(28):14241–65. doi: 10.1007/s11356-026-38177-x (PMC13582052; doi:10.1007/s11356-026-38177-x)
Supplement: Supplementary file 1 [file 11356_2026_38177_MOESM1_ESM.docx]

**Integrated physical-chemical remediation and human health risk assessment of multi-metal contaminated industrial soils**

Lucia Marsich^a*^ Alessio Ferluga^b^, Luca Cozzarini^a^, Paolo Bevilacqua^a^

^a^ Department of Engineering and Architecture University of Trieste Via Valerio 10, Trieste I-34127, Italy

^b^Research & Innovation Department, MaterialScan Srl., Via Capodistria 28, Trieste I-34145, Italy

^*^Corresponding author: Lucia Marsich, e-mail: lmarsich@units.it

**Supplementary material**

Table 1. Concentration distribution of heavy metals according to particle size (mg/kg).

| **Sieve size range (mm)** | Sb  (mg/kg) | As  (mg/kg) | Cd  (mg/kg) | Hg  (mg/kg) | Pb  (mg/kg) | Cu  (mg/kg) | Se  (mg/kg) | Tl  (mg/kg) | Zn  (mg/kg) |
| --- | --- | --- | --- | --- | --- | --- | --- | --- | --- |
| -0.010 | 225 ± 26 | 715 ± 79 | 7570 ± 696 | 252 ± 40 | 48000 ± 5520 | 2150 ± 159 | 383 ± 26 | 27.9 ± 2.1 | 99400 ± 6859 |
| -0.075 +0.010 | 196 ± 23 | 637 ± 70 | 2340 ± 215 | 103 ± 16.2 | 52600 ± 6049 | 1520 ± 113 | 185 ± 13 | 12.2 ± 0.9 | 59300 ± 4092 |
| -0.150 +0.075 | 80.7 ± 9.4 | 127 ± 14 | 594 ± 55 | 28.9 ± 4.5 | 13600 ± 1564 | 514 ± 38 | 37.9 ± 2.6 | 10.4 ± 0.8 | 23000 ± 1587 |
| -0.255 +0.150 | 22.9 ± 2.7 | 31.0 ± 3.4 | 171 ± 16 | 3.2 ± 0.5 | 3760 ± 432 | 137 ± 10 | 8.8 ± 0.6 | 3.7 ± 0.3 | 6520 ± 450 |
| -0.841 +0.255 | 32.3 ± 3.8 | 47.0 ± 5.2 | 293 ± 27 | 5.3 ± 0.8 | 4250 ± 489 | 224 ±17 | 7.5 ± 0.5 | 3.2 ± 0.2 | 7310 ± 504 |
| -4.76 +0.841 | 72.7 ± 8.5 | 140 ± 15 | 787 ± 72 | 27.5 ± 4.3 | 10800 ± 1242 | 419 ± 31 | 40.3 ± 2.8 | 7.1 ± 0.5 | 23700 ± 1635 |
| -19 +4.76 | 182 ± 21 | 189 ± 21 | 1840 ± 169 | 30.2 ± 4.7 | 24100 ± 2772 | 628 ± 46 | 34.2 ± 2.4 | 10.4 ± 0.8 | 42200 ± 2912 |
| -36 +19 | 99.1 ± 11.6 | 104 ± 14 | 308 ± 28 | 13.4 ± 2.1 | 10400 ± 1196 | 559 ± 41 | 11.1 ± 0.8 | 7.6 ± 0.6 | 73400 ± 5465 |
| +36 | 42.1 ± 4.9 | 97 ± 11 | 905 ± 83 | 30.5 ± 4.8 | 7030 ± 809 | 257 ± 19 | 42.2 ± 2.9 | 23.2 ± 1.8 | 24100 ± 1663 |

Table 2. Summary of hazard quotients and indexes of the -4.76 +0.075 mm soil fraction for non-residential (outdoor worker) and residential (adult and child) exposure scenarios. Values greater than unity are highlighted in bold.

|  | Outdoor worker | | | | Residential Adult | | | | Residential Child | | | |
| --- | --- | --- | --- | --- | --- | --- | --- | --- | --- | --- | --- | --- |
|  | HQ_ing_ | HQ_derm_ | HQ_inh_ | HI | HQ_ing_ | HQ_derm_ | HQ_inh_ | HI | HQ_ing_ | HQ_derm_ | HQ_inh_ | HI |
| **Sb** | 1.0E-01 | 4.5E-02 | 7.1E-05 | 1.5E-01 | 1.7E-01 | 4.5E-02 | 1.1E-04 | 2.1E-01 | **1.6E+00** | 2.9E-01 | 5.4E-04 | **1.9E+00** |
| **As** | 2.5E-01 | 4.9E-02 | 2.5E-03 | 3.0E-01 | 4.0E-01 | 4.8E-02 | 4.1E-03 | 4.5E-01 | **3.7E+00** | 3.1E-01 | 1.9E-02 | **4.0E+00** |
| **Cd** | **5.1E+00** | **1.4E+00** | 2.6E-02 | **6.5E+00** | **8.3E+00** | **1.3E+00** | 4.3E-02 | **9.7E+00** | **7.8E+01** | **8.7E+00** | 2.0E-01 | **8.7E+01** |
| Cr | 2.6E-05 | 1.3E-04 | n.a. | 1.6E-04 | 4.2E-05 | 1.3E-04 | n.a. | 1.7E-04 | 4.0E-04 | 8.5E-04 | n.a. | 1.3E-03 |
| Co | 2.7E-02 | 1.8E-03 | 6.9E-04 | 2.9E-02 | 4.3E-02 | 1.7E-03 | 1.1E-03 | 4.6E-02 | 4.0E-01 | 1.1E-02 | 5.2E-03 | 4.2E-01 |
| Cu | 7.9E-03 | 5.2E-04 | n.a. | 8.4E-03 | 1.3E-02 | 5.1E-04 | n.a. | 1.3E-02 | 1.2E-01 | 3.3E-03 | n.a. | 1.2E-01 |
| Fe | 2.5E-02 | 1.6E-03 | n.a. | 2.7E-02 | 4.0E-02 | 1.6E-03 | n.a. | 4.2E-02 | 3.8E-01 | 1.1E-02 | n.a. | 3.9E-01 |
| **Hg** | 1.0E-01 | 8.7E-03 | 5.4E-05 | 1.1E-01 | 1.7E-01 | 8.5E-03 | 8.8E-05 | 1.8E-01 | **1.6E+00** | 5.6E-02 | 4.1E-04 | **1.6E+00** |
| Ni | 1.7E-03 | 2.8E-03 | 4.8E-04 | 4.9E-03 | 2.7E-03 | 2.7E-03 | 7.7E-04 | 6.2E-03 | 2.5E-02 | 1.8E-02 | 3.6E-03 | 4.7E-02 |
| Se | 9.7E-03 | 8.0E-04 | 1.2E-06 | 1.1E-02 | 1.6E-02 | 7.9E-04 | 2.0E-06 | 1.7E-02 | 1.5E-01 | 5.1E-03 | 9.5E-06 | 1.5E-01 |
| **Tl** | 7.4E-01 | 4.9E-02 | n.a. | 7.9E-01 | **1.2E+00** | 4.8E-02 | n.a. | **1.2E+00** | **1.1E+01** | 3.1E-01 | n.a. | **1.2E+01** |
| Sn | 3.6E-05 | 2.4E-06 | n.a. | 3.8E-05 | 5.8E-05 | 2.3E-06 | n.a. | 6.1E-05 | 5.4E-04 | 1.5E-05 | n.a. | 5.6E-04 |
| Ti | 8.2E-05 | 5.4E-06 | n.a. | 8.7E-05 | 1.3E-04 | 5.3E-06 | n.a. | 1.4E-04 | 1.2E-03 | 3.5E-05 | n.a. | 1.3E-03 |
| V | 3.0E-03 | 7.6E-03 | 7.8E-05 | 1.1E-02 | 4.8E-03 | 7.4E-03 | 1.3E-04 | 1.2E-02 | 4.5E-02 | 4.9E-02 | 5.9E-04 | 9.5E-02 |
| Zn | 5.1E-02 | 3.4E-03 | n.a. | 5.5E-02 | 8.3E-02 | 3.3E-03 | n.a. | 8.7E-02 | 7.8E-01 | 2.2E-02 | n.a. | 8.0E-01 |

Table 3. Summary of hazard quotients and indexes of the -4.76 + 0.075 mm non-magnetic soil fraction for non-residential (outdoor worker) and residential (adult and child) exposure scenarios. Values greater than unity are highlighted in bold. Hazard quotients and indexes for cobalt, nickel, and tin are not included because their concentrations were below the limit of quantification.

|  | Outdoor worker | | | | Residential Adult | | | | Residential Child | | | |
| --- | --- | --- | --- | --- | --- | --- | --- | --- | --- | --- | --- | --- |
|  | HQ_ing_ | HQ_derm_ | HQ_inh_ | HI | HQ_ing_ | HQ_derm_ | HQ_inh_ | HI | HQ_ing_ | HQ_derm_ | HQ_inh_ | HI |
| Sb | 1.8E-02 | 7.9E-03 | 1.2E-05 | 2.6E-02 | 2.9E-02 | 7.8E-03 | 2.0E-05 | 3.7E-02 | 2.7E-01 | 5.1E-02 | 9.3E-05 | 3.2E-01 |
| **As** | 6.2E-02 | 1.2E-02 | 6.4E-04 | 7.5E-02 | 1.0E-01 | 1.2E-02 | 1.0E-03 | 1.1E-01 | 9.4E-01 | 7.9E-02 | 4.8E-03 | **1.0E+00** |
| **Cd** | **2.1E+00** | 5.5E-01 | 1.1E-02 | **2.6E+00** | **3.4E+00** | 5.4E-01 | 1.7E-02 | **3.9E+00** | **3.1E+01** | **3.5E+00** | 8.1E-02 | **3.5E+01** |
| Cr | 3.5E-06 | 1.8E-05 | n.a. | 2.1E-05 | 5.7E-06 | 1.8E-05 | n.a. | 2.3E-05 | 5.3E-05 | 1.1E-04 | n.a. | 1.7E-04 |
| Cu | 1.2E-03 | 7.7E-05 | n.a. | 1.2E-03 | 1.9E-03 | 7.5E-05 | n.a. | 2.0E-03 | 1.8E-02 | 4.9E-04 | n.a. | 1.8E-02 |
| Fe | 6.1E-03 | 4.1E-04 | n.a. | 6.5E-03 | 1.0E-02 | 4.0E-04 | n.a. | 1.0E-02 | 9.3E-02 | 2.6E-03 | n.a. | 9.6E-02 |
| **Hg** | 9.0E-02 | 7.5E-03 | 4.7E-05 | 9.8E-02 | 1.5E-01 | 7.3E-03 | 7.5E-05 | 1.5E-01 | **1.4E+00** | 4.8E-02 | 3.5E-04 | **1.4E+00** |
| Se | 8.1E-03 | 6.7E-04 | 1.0E-06 | 8.8E-03 | 1.3E-02 | 6.6E-04 | 1.7E-06 | 1.4E-02 | 1.2E-01 | 4.3E-03 | 7.9E-06 | 1.3E-01 |
| **Tl** | 2.6E-01 | 1.7E-02 | n.a. | 2.8E-01 | 4.2E-01 | 1.7E-02 | n.a. | 4.4E-01 | **3.9E+00** | 1.1E-01 | n.a. | **4.0E+00** |
| Ti | 3.0E-05 | 2.0E-06 | n.a. | 3.2E-05 | 4.9E-05 | 1.9E-06 | n.a. | 5.1E-05 | 4.5E-04 | 1.3E-05 | n.a. | 4.7E-04 |
| V | 1.3E-03 | 3.3E-03 | 3.4E-05 | 4.6E-03 | 2.1E-03 | 3.2E-03 | 5.5E-05 | 5.4E-03 | 2.0E-02 | 2.1E-02 | 2.6E-04 | 4.1E-02 |
| Zn | 1.5E-02 | 1.0E-03 | n.a. | 1.7E-02 | 2.5E-02 | 1.0E-03 | n.a. | 2.6E-02 | 2.3E-01 | 6.6E-03 | n.a. | 2.4E-01 |

Table 4. Summary of hazard quotients and indexes after chemical extraction with 15 mM EDTA for non-residential (outdoor worker) and residential (adult and child) exposure scenarios. Values greater than unity are highlighted in bold. Hazard quotients and indexes for chromium, cobalt, nickel, tin, and vanadium are not included because their concentrations were below the limit of quantification.

|  | Outdoor worker | | | | Residential Adult | | | | Residential Child | | | |
| --- | --- | --- | --- | --- | --- | --- | --- | --- | --- | --- | --- | --- |
|  | HQ_ing_ | HQ_derm_ | HQ_inh_ | HI | HQ_ing_ | HQ_derm_ | HQ_inh_ | HI | HQ_ing_ | HQ_derm_ | HQ_inh_ | HI |
| Sb | 1.2E-02 | 5.1E-03 | 8.0E-06 | 1.7E-02 | 1.9E-02 | 5.0E-03 | 1.3E-05 | 2.4E-02 | 1.8E-01 | 3.3E-02 | 6.1E-05 | 2.1E-01 |
| As | 6.1E-02 | 1.2E-02 | 6.3E-04 | 7.4E-02 | 1.0E-01 | 1.2E-02 | 1.0E-03 | 1.1E-01 | 9.3E-01 | 7.8E-02 | 4.8E-03 | 1.0E+00 |
| **Cd** | **1.3E+00** | 3.5E-01 | 6.9E-03 | **1.7E+00** | **2.2E+00** | 3.5E-01 | 1.1E-02 | **2.5E+00** | **2.0E+01** | **2.3E+00** | 5.2E-02 | **2.3E+01** |
| Cu | 8.9E-04 | 5.8E-05 | n.a. | 9.4E-04 | 1.4E-03 | 5.7E-05 | n.a. | 1.5E-03 | 1.3E-02 | 3.8E-04 | n.a. | 1.4E-02 |
| Fe | 5.3E-03 | 3.5E-04 | n.a. | 5.7E-03 | 8.7E-03 | 3.5E-04 | n.a. | 9.0E-03 | 8.1E-02 | 2.3E-03 | n.a. | 8.3E-02 |
| Hg | 5.8E-02 | 4.8E-03 | 3.0E-05 | 6.2E-02 | 9.4E-02 | 4.7E-03 | 4.8E-05 | 9.8E-02 | 8.7E-01 | 3.1E-02 | 2.2E-04 | 9.0E-01 |
| Se | 5.0E-03 | 4.2E-04 | 6.5E-07 | 5.5E-03 | 8.2E-03 | 4.1E-04 | 1.1E-06 | 8.6E-03 | 7.6E-02 | 2.7E-03 | 4.9E-06 | 7.9E-02 |
| **Tl** | 2.2E-01 | 1.5E-02 | n.a. | 2.4E-01 | 3.6E-01 | 1.5E-02 | n.a. | 3.8E-01 | **3.4E+00** | 9.5E-02 | n.a. | **3.5E+00** |
| Ti | 2.4E-05 | 1.6E-06 | n.a. | 2.6E-05 | 3.9E-05 | 1.6E-06 | n.a. | 4.1E-05 | 3.7E-04 | 1.0E-05 | n.a. | 3.8E-04 |
| Zn | 1.3E-02 | 8.5E-04 | n.a. | 1.4E-02 | 2.1E-02 | 8.4E-04 | n.a. | 2.2E-02 | 2.0E-01 | 5.5E-03 | n.a. | 2.0E-01 |

Table 5. Summary of hazard quotients and indexes after chemical extraction with 30 mM EDTA for non-residential (outdoor worker) and residential (adult and child) exposure scenarios. Values greater than unity are highlighted in bold. Hazard quotients and indexes for antimony, chromium, cobalt, nickel, tin, and vanadium are not included because their concentrations were below the limit of quantification.

|  | Outdoor worker | | | | Residential Adult | | | | Residential Child | | | |
| --- | --- | --- | --- | --- | --- | --- | --- | --- | --- | --- | --- | --- |
|  | HQ_ing_ | HQ_derm_ | HQ_inh_ | HI | HQ_ing_ | HQ_derm_ | HQ_inh_ | HI | HQ_ing_ | HQ_derm_ | HQ_inh_ | HI |
| As | 5.4E-02 | 1.1E-02 | 5.6E-04 | 6.5E-02 | 8.8E-02 | 1.0E-02 | 9.0E-04 | 9.9E-02 | 8.2E-01 | 6.9E-02 | 4.2E-03 | 8.9E-01 |
| **Cd** | 6.8E-01 | 1.8E-01 | 3.5E-03 | 8.6E-01 | **1.1E+00** | 1.8E-01 | 5.7E-03 | **1.3E+00** | **1.0E+01** | **1.1E+00** | 2.6E-02 | **1.1E+01** |
| Cu | 7.3E-04 | 4.8E-05 | n.a. | 7.8E-04 | 1.2E-03 | 4.7E-05 | n.a. | 1.2E-03 | 1.1E-02 | 3.1E-04 | n.a. | 1.1E-02 |
| Fe | 5.3E-03 | 3.5E-04 | n.a. | 5.7E-03 | 8.6E-03 | 3.4E-04 | n.a. | 9.0E-03 | 8.0E-02 | 2.3E-03 | n.a. | 8.3E-02 |
| Hg | 5.5E-02 | 4.5E-03 | 2.8E-05 | 5.9E-02 | 8.8E-02 | 4.4E-03 | 4.6E-05 | 9.3E-02 | 8.3E-01 | 2.9E-02 | 2.1E-04 | 8.5E-01 |
| Se | 4.9E-03 | 4.0E-04 | 6.2E-07 | 5.3E-03 | 7.9E-03 | 3.9E-04 | 1.0E-06 | 8.3E-03 | 7.3E-02 | 2.6E-03 | 4.7E-06 | 7.6E-02 |
| **Tl** | 2.2E-01 | 1.4E-02 | n.a | 2.3E-01 | 3.5E-01 | 1.4E-02 | n.a | 3.6E-01 | **3.3E+00** | 9.1E-02 | n.a | **3.4E+00** |
| Ti | 2.4E-05 | 1.6E-06 | n.a. | 2.5E-05 | 3.8E-05 | 1.5E-06 | n.a. | 4.0E-05 | 3.6E-04 | 1.0E-05 | n.a. | 3.7E-04 |
| Zn | 1.1E-02 | 7.2E-04 | n.a. | 1.2E-02 | 1.8E-02 | 7.1E-04 | n.a. | 1.8E-02 | 1.7E-01 | 4.6E-03 | n.a. | 1.7E-01 |

Table 6. Summary of hazard quotients and indexes after chemical extraction with 45 mM EDTA for non-residential (outdoor worker) and residential (adult and child) exposure scenarios. Values greater than unity are highlighted in bold. Hazard quotients and indexes for antimony, chromium, cobalt, nickel, tin, and vanadium are not included because their concentrations were below the limit of quantification.

|  | Outdoor worker | | | | Residential Adult | | | | Residential Child | | | |
| --- | --- | --- | --- | --- | --- | --- | --- | --- | --- | --- | --- | --- |
|  | HQ_ing_ | HQ_derm_ | HQ_inh_ | HI | HQ_ing_ | HQ_derm_ | HQ_inh_ | HI | HQ_ing_ | HQ_derm_ | HQ_inh_ | HI |
| As | 5.0E-02 | 9.8E-03 | 5.1E-04 | 6.0E-02 | 8.0E-02 | 9.6E-03 | 8.3E-04 | 9.1E-02 | 7.5E-01 | 6.3E-02 | 3.9E-03 | 8.2E-01 |
| **Cd** | 4.8E-01 | 1.3E-01 | 2.5E-03 | 6.1E-01 | 7.8E-01 | 1.2E-01 | 4.0E-03 | 9.1E-01 | **7.3E+00** | 8.2E-01 | 1.9E-02 | **8.1E+00** |
| Cu | 6.2E-04 | 4.1E-05 | n.a. | 6.6E-04 | 1.0E-03 | 4.0E-05 | n.a. | 1.0E-03 | 9.4E-03 | 2.6E-04 | n.a. | 9.7E-03 |
| Fe | 5.3E-03 | 3.5E-04 | n.a. | 5.6E-03 | 8.6E-03 | 3.4E-04 | n.a. | 8.9E-03 | 8.0E-02 | 2.2E-03 | n.a. | 8.2E-02 |
| Hg | 4.8E-02 | 3.9E-03 | 2.5E-05 | 5.2E-02 | 7.8E-02 | 3.9E-03 | 4.0E-05 | 8.2E-02 | 7.2E-01 | 2.5E-02 | 1.9E-04 | 7.5E-01 |
| Se | 4.4E-03 | 3.6E-04 | 5.6E-07 | 4.7E-03 | 7.1E-03 | 3.5E-04 | 9.1E-07 | 7.4E-03 | 6.6E-02 | 2.3E-03 | 4.2E-06 | 6.8E-02 |
| **Tl** | 2.1E-01 | 1.4E-02 | n.a. | 2.3E-01 | 3.4E-01 | 1.4E-02 | n.a. | 3.6E-01 | **3.2E+00** | 9.0E-02 | n.a. | **3.3E+00** |
| Ti | 2.3E-05 | 1.5E-06 | n.a. | 2.4E-05 | 3.7E-05 | 1.5E-06 | n.a. | 3.9E-05 | 3.5E-04 | 9.7E-06 | n.a. | 3.6E-04 |
| Zn | 9.0E-03 | 5.9E-04 | n.a. | 9.6E-03 | 1.5E-02 | 5.8E-04 | n.a. | 1.5E-02 | 1.4E-01 | 3.8E-03 | n.a. | 1.4E-01 |

Table 7. Summary of hazard quotient and indexes after chemical extraction with 60 mM EDTA for non-residential (outdoor worker) and residential (adult and child) exposure scenarios. Values greater than unity are highlighted in bold. Hazard quotients and indexes for antimony, chromium, cobalt, nickel, tin, and vanadium are not included because their concentrations were below the limit of quantification.

|  | Outdoor worker | | | | Residential Adult | | | | Residential Child | | | |
| --- | --- | --- | --- | --- | --- | --- | --- | --- | --- | --- | --- | --- |
|  | HQ_ing_ | HQ_derm_ | HQ_inh_ | HI | HQ_ing_ | HQ_derm_ | HQ_inh_ | HI | HQ_ing_ | HQ_derm_ | HQ_inh_ | HI |
| As | 3.2E-02 | 6.3E-03 | 3.3E-04 | 3.8E-02 | 5.1E-02 | 6.2E-03 | 5.3E-04 | 5.8E-02 | 4.8E-01 | 4.0E-02 | 2.5E-03 | 5.2E-01 |
| **Cd** | 4.5E-01 | 1.2E-01 | 2.3E-03 | 5.7E-01 | 7.3E-01 | 1.2E-01 | 3.8E-03 | 8.5E-01 | **6.8E+00** | 7.7E-01 | 1.8E-02 | **7.6E+00** |
| Cu | 6.0E-04 | 4.0E-05 | n.a. | 6.4E-04 | 9.8E-04 | 3.9E-05 | n.a. | 1.0E-03 | 9.2E-03 | 2.6E-04 | n.a. | 9.4E-03 |
| Fe | 5.1E-03 | 3.3E-04 | n.a. | 5.4E-03 | 8.2E-03 | 3.3E-04 | n.a. | 8.6E-03 | 7.7E-02 | 2.1E-03 | n.a. | 7.9E-02 |
| Hg | 3.7E-02 | 3.1E-03 | 1.9E-05 | 4.0E-02 | 6.0E-02 | 3.0E-03 | 3.1E-05 | 6.3E-02 | 5.6E-01 | 2.0E-02 | 1.4E-04 | 5.8E-01 |
| Se | 4.2E-03 | 3.5E-04 | 5.4E-07 | 4.6E-03 | 6.8E-03 | 3.4E-04 | 8.8E-07 | 7.2E-03 | 6.4E-02 | 2.2E-03 | 4.1E-06 | 6.6E-02 |
| **Tl** | 2.0E-01 | 1.3E-02 | n.a. | 2.2E-01 | 3.3E-01 | 1.3E-02 | n.a. | 3.4E-01 | **3.1E+00** | 8.6E-02 | n.a. | **3.2E+00** |
| Ti | 2.3E-05 | 1.5E-06 | n.a. | 2.4E-05 | 3.7E-05 | 1.5E-06 | n.a. | 3.8E-05 | 3.4E-04 | 9.6E-06 | n.a. | 3.5E-04 |
| Zn | 8.7E-03 | 5.8E-04 | n.a. | 9.3E-03 | 1.4E-02 | 5.6E-04 | n.a. | 1.5E-02 | 1.3E-01 | 3.7E-03 | n.a. | 1.4E-01 |

Table 8. Summary of hazard quotient and indexes after chemical extraction with 75 mM EDTA for non-residential (outdoor worker) and residential (adult and child) exposure scenarios. Values greater than unity are highlighted in bold. Hazard quotients and indexes for antimony, chromium, cobalt, nickel, tin, and vanadium are not included because their concentrations were below the limit of quantification.

|  | Outdoor worker | | | | Residential Adult | | | | Residential Child | | | |
| --- | --- | --- | --- | --- | --- | --- | --- | --- | --- | --- | --- | --- |
|  | HQ_ing_ | HQ_derm_ | HQ_inh_ | HI | HQ_ing_ | HQ_derm_ | HQ_inh_ | HI | HQ_ing_ | HQ_derm_ | HQ_inh_ | HI |
| As | 2.8E-02 | 5.5E-03 | 2.9E-04 | 3.4E-02 | 4.5E-02 | 5.4E-03 | 4.7E-04 | 5.1E-02 | 4.2E-01 | 3.5E-02 | 2.2E-03 | 4.6E-01 |
| **Cd** | 4.3E-01 | 1.1E-01 | 2.2E-03 | 5.4E-01 | 6.9E-01 | 1.1E-01 | 3.6E-03 | 8.1E-01 | **6.5E+00** | 7.3E-01 | 1.7E-02 | **7.2E+00** |
| Cu | 5.8E-04 | 3.8E-05 | n.a. | 6.2E-04 | 9.4E-04 | 3.8E-05 | n.a. | 9.8E-04 | 8.8E-03 | 2.5E-04 | n.a. | 9.0E-03 |
| Fe | 4.7E-03 | 3.1E-04 | n.a. | 5.0E-03 | 7.7E-03 | 3.1E-04 | n.a. | 8.0E-03 | 7.2E-02 | 2.0E-03 | n.a. | 7.4E-02 |
| Hg | 3.6E-02 | 3.0E-03 | 1.9E-05 | 3.9E-02 | 5.9E-02 | 2.9E-03 | 3.0E-05 | 6.2E-02 | 5.5E-01 | 1.9E-02 | 1.4E-04 | 5.7E-01 |
| Se | 3.1E-03 | 2.6E-04 | 4.0E-07 | 3.4E-03 | 5.1E-03 | 2.5E-04 | 6.5E-07 | 5.3E-03 | 4.7E-02 | 1.7E-03 | 3.1E-06 | 4.9E-02 |
| **Tl** | 1.8E-01 | 1.2E-02 | n.a. | 1.9E-01 | 2.9E-01 | 1.1E-02 | n.a. | 3.0E-01 | **2.7E+00** | 7.5E-02 | n.a. | **2.7E+00** |
| Ti | 2.2E-05 | 1.5E-06 | n.a. | 2.4E-05 | 3.6E-05 | 1.4E-06 | n.a. | 3.7E-05 | 3.3E-04 | 9.4E-06 | n.a. | 3.4E-04 |
| Zn | 8.0E-03 | 5.3E-04 | n.a. | 8.6E-03 | 1.3E-02 | 5.2E-04 | n.a. | 1.4E-02 | 1.2E-01 | 3.4E-03 | n.a. | 1.3E-01 |
